# Unraveling the Spiraling Radiation: A Phylogenomic Analysis of Neotropical *Costus* L

**DOI:** 10.3389/fpls.2020.01195

**Published:** 2020-08-14

**Authors:** Eugenio Valderrama, Chodon Sass, Maria Pinilla-Vargas, David Skinner, Paul J. M. Maas, Hiltje Maas-van de Kamer, Jacob B. Landis, Clarice J. Guan, Chelsea D. Specht

**Affiliations:** ^1^ School of Integrative Plant Science, Section of Plant Biology and the L.H. Bailey Hortorium, Cornell University, Ithaca, NY, United States; ^2^ The University and Jepson Herbaria, University of California, Berkeley, Berkeley, CA, United States; ^3^ Le Jardin Ombragé, Tallahassee, FL, United States; ^4^ Section Botany, Naturalis Biodiversity Center, Leiden, Netherlands

**Keywords:** Costaceae, Zingiberales, plant radiation, phylogenomics, *Costus*, Neotropical

## Abstract

The family of pantropical spiral gingers (Costaceae Nakai; *c.* 125 spp.) can be used as a model to enhance our understanding of the mechanisms underlying Neotropical diversity. Costaceae has higher taxonomic diversity in South and Central America (*c.* 72 Neotropical species, *c.* 30 African, *c.* 23 Southeast Asian), particularly due to a radiation of Neotropical species of the genus *Costus* L. (*c.* 57 spp.). However, a well-supported phylogeny of the Neotropical spiral gingers including thorough sampling of proposed species encompassing their full morphologic and geographic variation is lacking, partly due to poor resolution recovered in previous analyses using a small sampling of loci. Here we use a phylogenomic approach to estimate the phylogeny of a sample of Neotropical *Costus* species using a targeted enrichment approach. Baits were designed to capture conserved elements’ variable at the species level using available genomic sequences of *Costus* species and relatives. We obtained 832 loci (generating 791,954 aligned base pairs and 31,142 parsimony informative sites) for samples that encompassed the geographical and/or morphological diversity of some recognized species. Higher support values that improve the results of previous studies were obtained when including all the available loci, even those producing unresolved gene trees and having a low proportion of variable sites. Concatenation and coalescent-based species trees methods converge in almost the same topology suggesting a robust estimation of the relationships, even under the high levels of gene tree conflict presented here. The bait set design here presented made inferring a robust phylogeny to test taxonomic hypotheses possible and will improve our understanding of the origins of the charismatic diversity of the Neotropical spiral gingers.

## Introduction

One of the most widely recognized patterns in ecology and biogeography is that lineages tend toward species richness in tropical regions (Kreft and Jetz, 2007); however, the mechanisms that originate such patterns of diversity are still poorly understood. In addition, richness is not uniform across the tropical regions; the Neotropics stand as the most diverse with around 90,000–110,000 species of seed plants that could exceed the numbers of tropical Africa with 30,000–35,000 spp. and tropical Asia and Oceania with 40,000–82,000 spp., combined (Antonelli and Sanmartín, 2011; Hughes et al., 2013). Hypotheses addressing higher species richness in the Neotropics include opportunities for allopatric speciation, the availability of new habitats through uplift of the Andes (Gentry, 1982), major habitat and climate shifts prompted by shifts in the Amazon river drainage (Hoorn et al., 2010), and closure of the Panama isthmus (Bacon et al., 2013). Possibilities for prezygotic reproductive isolation driven by shifts in pollination syndromes (Serrano-Serrano et al., 2017), adaptation to local conditions leading to ecological speciation (Antonelli et al., 2018), or the effects of polyploidization on diversification rates (Soltis and Soltis, 2009; Landis et al., 2018) of Neotropical lineages are additional mechanisms that could explain the relatively higher diversity of Neotropical plant lineages compared to their Paleotropical congeners. Alternative explanations for the uneven distribution of biodiversity at continental scale include dispersal dynamics driven by historical changes in climate and differential extinction rates (Meseguer and Condamine, 2020). Specifically, the importance of extinction has been discussed to understand lower species richness in Africa compared to the Neotropics and South-East Asia (Couvreur, 2015).

The idea of the importance of interactions with pollinators for the diversification of flowering plants traces back to Darwin (1862). Selection can act to mold the characteristics of flowers driven by their predominant or most effective pollinators (Stebbins, 1970). The combination of traits (*e.g.* morphology, color, scent, size, rewards) associated with particular pollinator groups are known as pollination syndromes (Faegri and Pijl, 1979; Rosas-Guerrero et al., 2014). A recent study suggests that floral traits related to pollination efficiency (flower shape and orientation, position of reproductive organs) could be more important than widely considered traits including exposure, display size, scent, color, symmetry, and timing of anthesis (Dellinger et al., 2019). Although the validity of the concept of pollination syndromes has been debated, studies have been able to predict pollinators using floral traits and to confirm a stronger association in plants distributed in the tropics and associated with bats, bees, and hummingbirds (Rosas-Guerrero et al., 2014; Ashworth et al., 2015). Diversification rates within hummingbird pollinated lineages have been shown to be higher than in bee pollinated ones (Lagomarsino et al., 2016; Serrano-Serrano et al., 2017) and shifts towards hummingbird pollination syndrome associated with areas of high diversity of these birds in the Neotropics (Tripp and Manos, 2008). Furthermore, although syndromes can constitute specialized systems on specific pollinator guilds, they have been shown to be labile, with transitions and reversions happening repeatedly through the history of some Neotropical plant lineages (Tripp and Manos, 2008).

The family of pantropical spiral gingers (Costaceae Nakai; *c.* 125 spp.) can be used as a model to enhance our understanding of the mechanisms underlying Neotropical diversity. Costaceae has higher taxonomic diversity in South and Central America (*c.* 72 Neotropical species, *c.* 30 African, *c.* 23 Southeast Asian), particularly due to a radiation of Neotropical species of the genus *Costus* L. (*c.* 57 spp.). *Costus* is broadly distributed in the New World inhabiting lowland rain forest, montane rain forests, and periodically inundated várzea forests in elevations from the sea level up to 2,000 m, but mainly below 1,000 m (Maas, 1972). Previous studies have shown that the Neotropical species of *Costus* show multiple shifts in pollination syndromes, with closely related species that are associated with either insects or birds demonstrating rapid ecological isolation (Kay et al., 2005; Specht et al., 2012; Salzman et al., 2015). Furthermore, species within the Neotropical *Costus* clade have shown higher diversification rates during the last *c.* 10–20 million years (see André et al., 2016 for a discussion on the dates) as compared with the rest of the family, including the closely related African *Costus* lineages, and the prevalence in these lineages of sympatric species is higher regardless of time to differentiate (André et al., 2016). However, attempts to estimate phylogenies with a handful of plastid and nuclear loci have led to unresolved relationships in the species-rich clade comprising the Neotropical *Costus* (Salzman et al., 2015; André et al., 2016). Therefore, a well-supported phylogeny of the Neotropical spiral gingers, including thorough sampling of proposed species encompassing their full morphologic and geographic variation, is much needed.

The low resolution in the phylogenies adds uncertainty to the current understanding of the mechanisms that produced the charismatic and intriguing diversity within the spiral gingers. For example, a clear understanding of the phylogenetic relationships of closely related species that have undergone major shifts in morphology would allow us to test the genetic mechanisms underlying the changes between ornithophilous (bird attracting) and melittophilous (bee attracting) pollination syndromes that repeatedly took place in the history of this lineage and to characterize the role of these genetic mechanisms in shaping the speciation processes (Salzman et al., 2015). In addition, a fully resolved phylogeny of the species-rich clades of Costaceae would enlighten the taxonomy of the group (Maas, 1972; Maas, 1977), with extensive implications for understanding spatial and temporal patterns of distribution.

The difficulties in estimating robust, species-level phylogenies for speciose lineages are expected because of the combination of processes affecting recent radiations, including incomplete lineage sorting due to rapid differentiation and/or large population sizes and hybridization followed by introgression (Pamilo and Nei, 1988; Maddison, 1997; Maddison and Knowles, 2006). Coupled with the advances in sequencing technologies (Lemmon and Lemmon, 2013; McCormack and Faircloth, 2013), target enrichment provides a solution for the need to acquire the hundreds or thousands of loci throughout the genome that are necessary to unveil the phylogenies of species rich and recently radiated plant lineages (Cronn et al., 2012). This is particularly true for those groups with large genome sizes, for which the sequencing and computational costs associated with whole-genome approaches quickly become restrictive as accession numbers increase (McKain et al., 2018). One of the additional and major advantages of targeted sequencing is that fragmented DNA from herbarium specimens can be used successfully (Hart et al., 2016; Brewer et al., 2019) allowing the sampling of lineages that are only available as herbarium specimens and to include specimens representing historic distributions. The accessions available for phylogenetic studies in natural history collections are essential to survey the diversity of species-rich groups, to include narrow endemics difficult to collect in the field and to account for variation in widespread and polymorphic species (Särkinen et al., 2012; Buerki and Baker, 2016; Bieker and Martin, 2018; Valderrama et al., 2018). The use of target enrichment strategies to gather low or single copy nuclear loci for phylogenomics of plant lineages at different scales (Nicholls et al., 2015; Sass et al., 2016) is becoming a standard technique, and the establishment of universal probe sets could reduce costs and time while enabling the merging of datasets from different studies and across plant lineages (Johnson et al., 2019; Larridon et al., 2020). However, divergence between the target sequences and the baits does affect capture efficiency (Larridon et al., 2020). The alternative process of designing custom baits allows researchers to aim for variable loci at the specific taxonomic scale of interest for the focus group, provided preliminary data is available for bait design (McKain et al., 2018). The increasing availability of genomic and transcriptomic data across the tree of life and the accessibility of pipelines to identify potential orthologs with low or single copy number (Chamala et al., 2015; Faircloth, 2016) help support the design of clade-specific bait sets (*e.g.* Vatanparast et al., 2018; Finch et al., 2019; Soto Gomez et al., 2019). Larridon et al. (2020) compared family specific probes and the Angiosperms-353 (Johnson et al., 2019) and obtained similar results with both approaches. However, universal probes could save labor and allow merging datasets of multiple studies, while taxa specific probes could improve recovery of target loci.

Here we use a phylogenomic approach to estimate the phylogeny of the Neotropical species of *Costus*, using a targeted enrichment approach. Baits were designed to capture conserved elements as identified from genomic sequences of *Costus* species and relatives. We sampled described and newly proposed species to test for reciprocal monophyly and included multiple samples from widespread and enigmatic species covering observed morphologic and geographic variation. DNA was extracted from living collections, field collected material, and herbarium samples to include population-level diversity. The resulting phylogeny of the Neotropical spiral gingers sheds light on the taxonomy of this lineage and enables us to confirm the multiple shifts in pollination syndromes during the evolution of *Costus* species.

## Materials and Methods

### Taxon Sampling

Samples were chosen such that, when possible, they encompassed the geographical and/or morphological diversity of each species recognized or proposed for an updated monograph (Maas, 1972; Maas, 1977; Maas et al. pers. comm.). Widely distributed species or those being tested for monophyly include up to four accessions representing geographic and/or phenotypic variation. For field collected specimens, DNA was extracted from silica-dried leaf material, and voucher specimens were deposited in herbaria or in living collections (see **Supplementary Table 1**). For those not vouchered or in cultivation but included to increase geographic sampling for a given species, provenance data is recorded on inaturalist.org and is cross referenced with accession numbers. In total, thirty-one of *c.* 57 Neotropical *Costus* species were included in this analysis with sampling from field and herbarium-collected material.

### Baits Design

Bait design followed the phyluce pipeline (Faircloth et al., 2012; Faircloth, 2016) with the following modifications. Instead of using annotated genomes and generating simulated reads from the assembled genomes, raw Illumina reads from *Costus spicatus* (Jacq.) Sw. and *Costus longibracteolatus* Maas genomic data (unpublished, Ana M.R. Almeida) were cleaned with TrimGalore 0.6.0 (Martin, 2011; https://github.com/FelixKrueger/TrimGalore) using a size cutoff of 36 bp (–length 36) and used in the alignment step. For the 7,723 regions that were found in the phyluce pipeline, local *de novo* assembly was performed with aTRAM 2.0 (Allen et al., 2018) using the cleaned *Costus* reads for each species separately, using two *de novo* assembly algorithms—Velvet 1.2.10 (Zerbino and Birney, 2008) and SPAdes 3.11.1 (Bankevich et al., 2012). Regions which generated a single *de novo* assembly contig after merging overlapping contigs (4-FinalAssembly.pl by Sonal Singhal; https://github.com/CGRL-QB3-UCBerkeley/denovoTargetCapturePopGen/blob/master/4-FinalAssembly) were carried on to subsequent filtering steps (2,686 regions). All regions that were found as a single contig in either *Costus* genome were carried forward; if the same region was found in both *Costus* genomes, the longer of the two regions was chosen. Several steps were added to the phyluce pipeline to filter regions of repetitive or putatively nonhomologous regions and to expand the dataset to regions that had known overlap with other published studies in the Zingiberales. 1) Sequences shorter than 160 bp were removed [2,388 regions remained]. 2) megaBLAST (Morgulis et al., 2008) all against all was conducted, and sequences which matched to any region other than itself were removed [removed 619 regions]. 3) BLAST (Altschul et al., 1990) searches against monocot mitochondrial and plastid genomes downloaded from the RefSeq database (O’Leary et al., 2016) were performed to remove sequences that matched these genomes [removed 399 regions]. At this point 2,019 regions passed filtering. 4) BLAST analyses to the RepeatMasker database (Smit et al., 2015) were used to identify regions matching to transposons [removed five regions]. 5) Only regions with a GC content between 37 and 55% GC were retained to improve bait capture efficiency [removed two regions]. 6) Baits from a single *Costus* representative found in Sass et al. (2016) were added to the set [240 regions added]. 7) bait regions that were generated as part of Carlsen et al. (2018) were subjected to local *de novo* assembly with aTRAM as described above, to find these bait regions for *Costus* [47 regions added after filtering for length and GC content, as above]. Some regions were added that are of specific interest for studies addressing development and morphological characters (note: these were excluded from the downstream analyses of the present study) for a total target length of approximately 1 million base pairs. This dataset was used to create custom 100 mer probes in a 20 K design by myBaits (Arbor Biosciences, Ann Arbor, MI, USA) with 3× tiling.

### DNA Extraction and Library Preparation

Leaf material was dried *in silica* and extracted using an SDS protocol (Edwards et al., 1991; Konieczny and Ausubel, 1993). Zymo DNA Clean & Concentrator-5 kits were used to purify the extractions (Zymo Research, Irvine, CA, USA). The size of the obtained fragments was checked in a 1% agarose gel. When average fragment size was above 350 bp, we followed the manufacturer’s protocol for the Covaris E220 evolution Focused-ultrasonicator (Covaris, Woburn, MA, USA) to obtain an average fragment size of 350 bp. Double-sided-size selection was performed with size selection beads using a homemade solution of Carboxyl-modified Sera-Mag Magnetic Speed-beads (Thermo Fisher Scientific, Freemont, CA) in a PEG/NaCl buffer (Rowan et al., 2017).

Dual-indexed libraries were prepared following manufacturer’s recommendations with the KAPA Hyper Prep kit with 500 ng of size-selected DNA quantified with Qubit 3.0 Fluorometer (Life Technologies, Grand Island, NY, USA). The volume per reaction was reduced to 1/5th following the recommendations of Lydia Smith at the Evolutionary Genetics Laboratory at UC Berkeley (comm. pers.; protocol available at https://osf.io/fkj2x). We used TruSeq style barcodes (8 bp) with a Stubby Adapter (see the **Supplementary Material Data**) and indexing primers provided by the Vincent J. Coates Genomics Sequencing Laboratory at UC Berkeley. Indexed samples were pooled (4–10 samples/reaction) and enriched with the custom probes following the manufacturer’s instructions (myBaits Manual v4.01, Arbor Biosciences, Ann Arbor, MI, USA) with a hybridization temperature of 65°C for 24 h. Because different blocking oligos show significant differences in performance (Portik et al., 2016), we used the Roche Universal Blocking Oligo Kit and SeqCap EZ Developer Reagent with plant C0t-1 DNA instead of the Blockers Mix supplied with the baits. Capture efficiency was assessed by comparing the amplification of target and off-target regions with a qPCR using the PowerUp™ SYBR™ Green Master Mix (Thermo Fisher Scientific Baltics UAB, Vilnius, Lithuania) in the ViiA 7 Real-Time PCR System (Applied Biosystems, Foster City, CA, USA). The enriched and pooled libraries (100 individuals in 11 reactions) were sequenced on a lane of NovaSeq SP 150PE in the Vincent J. Coates Genomics Sequencing Laboratory at UC Berkeley.

### Reads Processing, Assembly and Alignment

Reads were trimmed to remove low quality bases and adapter sequences with TrimGalore and normalized to 100× coverage using BBNorm (BBMap 38.74; Bushnell, 2020). HybPiper 1.3.1 (Johnson et al., 2016) with default settings was used to extract the reads that were mapped to the 1,521 target loci with BWA 0.7.12 (Li and Durbin, 2009). Mapped reads were assembled into contigs with SPAdes 3.13.1 (Bankevich et al., 2012) and discarded when coverage was lower than 8×. Summary statistics of the mapped reads were obtained with samtools 1.3 (Li et al., 2009). Only exonic sequences were kept in the downstream analyses to avoid inaccurate alignments. Paralog sequences for the assembled loci were retrieved with HybPiper. Loci with paralog warnings obtained for more than 5% of the accessions with recovered loci were excluded from downstream analyses. Available chloroplast genomes (Sass et al., 2016) were used to assemble plastid coding sequences using HybPiper and aTRAM; however, we recovered a very low amount of off-target reads in our libraries preventing us from generating comparable plastid sequences for our accessions. Contigs obtained were aligned using MAFFT 7.271 (Katoh and Toh, 2010) with the iterative (maximum iterations set to 10,000) refinement method incorporating local pairwise alignment information and with a gap opening penalty of 10. Trimal 1.3 (Capella-Gutiérrez et al., 2009) was used to remove poorly aligned bases and spurious sequences (-resoverlap and -seqoverlap parameters, 0.75. and 75 respectively).

### Phylogenetic Inference

The alignments were used to estimate gene trees for each locus using RAxML 8.2.12 (Stamatakis, 2014) with the rapid bootstrap analysis (200 replicates) and search for best-scoring maximum likelihood tree in the same run with a GTR + GAMMA substitution model. Abnormally long branches were determined by TreeShrink (Mai and Mirarab, 2018) with default values for the species mode (*α* =0.05, b = 5%). The algorithm estimates the distribution of branch lengths for each individual within the gene trees and uses it to identify significantly long branches and removes them in the respective trees and alignments.

We concatenated the loci and fitted a GTR + GAMMA substitution model for each gene and allowed IQ-Tree 1.6.10 (Nguyen et al., 2015; Chernomor et al., 2016; Kalyaanamoorthy et al., 2017) to explore merging those partitions corresponding to each gene using the greedy heuristic algorithm (Lanfear et al., 2012) before finding trees. The analysis became computationally intractable when considering the many possible schemes to merge the partitions of so many genes. We therefore used the relaxed cluster algorithm (rcluster option; Lanfear et al., 2014) that examines only the top 10% of the partition merging schemes. To assess the impact of using the relaxed cluster over the greedy heuristic algorithm, we also reduced the number of genes dividing the loci into three subsets to complete more thorough analyses using the greedy algorithm. Focusing on nodes with higher support within each gene tree (due to the overall low support values for individual gene trees), we used 40, 50, and 60% as threshold values of the upper quartile of rapid bootstrap support values obtained in RAxML for each gene tree to subset the obtained loci. This enabled us to focus on the loci that produced better supported trees and could potentially be more informative for our study.

We used ultrafast bootstrap approximation (Hoang et al., 2018) combined with the single branch SH-like approximate likelihood ratio test (SH-aLRT; Guindon et al., 2010) implemented in IQ-Tree, each with 10,000 replicates to assess the support of the resulting trees. The ultrafast bootstrap support values resulting from the analyses with the different subsets were mapped to the topology obtained with all loci using phangorn 2.5.5 (Schliep, 2011). Differences among subsets in ultrafast bootstrap support values were tested with a Friedman test (Friedman, 1937) and *post hoc* Wilcoxon signed-rank tests (Wilcoxon, 1945) with a Bonferroni correction (Bonferroni, 1935) in R 3.5.1 (R Core Team, 2013). Whenever possible, analyses were run in the CIPRES portal (Miller et al., 2011).

To consider incongruence among gene trees using methods statistically consistent under a multispecies coalescent model, we estimated species trees with ASTRAL 5.6.3 (Zhang et al., 2017) with all the obtained loci and the subsets. We contracted the low support branches of the gene trees (<10%) to improve the accuracy of the method (Zhang et al., 2017) using Newick Utilities 1.6 (Junier and Zdobnov, 2010). R packages treeio 1.10.0 and ggtree 2.0.4 (Yu et al., 2017) were used to plot the quartet support values estimated with ASTRAL on the resulting topology using the −t2 output option. We used phytools 0.6-99 (Revell, 2012) function cophylo to visually compare the concatenation and coalescent-based species trees.

Preliminary analysis indicated that the accessions from other Neotropical genera (*Dimerocostus* Kuntze and *Chamaecostus* C. Specht & D. W. Stev) were very divergent compared to the differentiation found within the Neotropical *Costus* lineages and could inflate the tree diameter and reduce the ability of TreeShrink to detect abnormally long branches, so only *Costus* species were included in the final analyses, with the African *C. fenestralis* Maas & H.Maas used as an outgroup based on previous studies confirming that Neotropical *Costus* are derived from African lineages (Salzman et al., 2015; André et al., 2016). Alignments with too few individuals (<50) and subsequently, individuals with too few loci (<520 for the analysis with all the obtained loci) were excluded from the analyses to avoid the effects of excessive missing data. Whenever necessary, accessions were removed from the alignments using AMAS 0.98 that was also used to generate summary statistics (Borowiec, 2016). The proportion of parsimony informative sites was compared among subsets with a Fisher–Pitman permutation test implemented in the R package coin 1.3-1 (Hothorn et al., 2008) using an approximative (Monte Carlo) reference distribution with 100,000 replicates and a *post hoc* pairwise permutation test with a Bonferroni correction to adjust *p* values for multiple comparisons with rcompanion 2.3.25 package (Mangiafico, 2016). Because of the assumed absence of hybridization and introgression transversal to the phylogenetic inference methods, all analyses were remade excluding the individuals identified as potential hybrids to avoid their impact on the results. The potential hybrids (nine individuals) and candidate parentals were identified based on morphological characters, and access to detailed images of those individuals is provided in **Supplementary Table 1**. We also estimated an evolutionary network for the New World *Costus* species using the NeighborNet algorithm with uncorrected p-distances and 500 bootstrap replicates in SplitsTree 4.16.1 (Huson and Bryant, 2005).

### Phylogenetic Comparative Methods

To better understand the evolution of pollination syndromes in the Neotropical *Costus* clade we used stochastic character mapping (Huelsenbeck et al., 2003) to reconstruct ancestral character states. Taxa were coded as either bee pollinated (melittophilous) or bird pollinated (ornithophilous) based on their morphological display of pollination syndrome. We used models with equal and different transition rates for the shifts in pollination syndromes, as implemented in phytools, and generated 1,000 stochastic character maps with the resulting phylogeny of the concatenation approach. The equal and different rate models were compared with a likelihood-ratio test. Individuals of the same species that formed monophyletic clades were pruned from the phylogeny leaving a single accession per species. The resulting character maps were summarized to estimate posterior probabilities of the ancestral pollination syndromes of *Costus* diversity in the new world tropics. To explore biogeographical history of the study group, we assigned species to the World Wildlife Fund’s ecoregions (Olson et al., 2001) as summarized by Antonelli et al. (2018). We used the data presented by Salzman et al. (2015) and from herbaria records available in the Global Biodiversity Information Facility to assign the areas to the species. Undescribed taxa and poorly known lineages were excluded to avoid underestimating the distribution ranges. Nonmonophyletic species were reduced to a single accession by keeping the one that matched the known phylogenetic affinities (Salzman et al., 2015; André et al., 2016). We used BioGeoBEARS likelihood framework to fit a model of Dispersal-Extinction Cladogenesis (DEC) to our dataset (Matzke, 2013), allowing any species to occupy a maximum of six areas of the eight included in the analysis. To fit a DEC model the tree was forced to be ultrametric using penalized likelihood with correlated rate variation among branches (Kim and Sanderson, 2008) using the chronos function of ape R package (Paradis and Schliep, 2019), and branch lengths were multiplied by 100,000 to have a range of values between 1 and 1,000. The +J model was not considered in the analysis because of its conceptual and statistical flaws (Ree and Sanmartín, 2018).

## Results

### Capture Efficiency and Phylogenetic Information of Captured Reads

**Figure 1:**
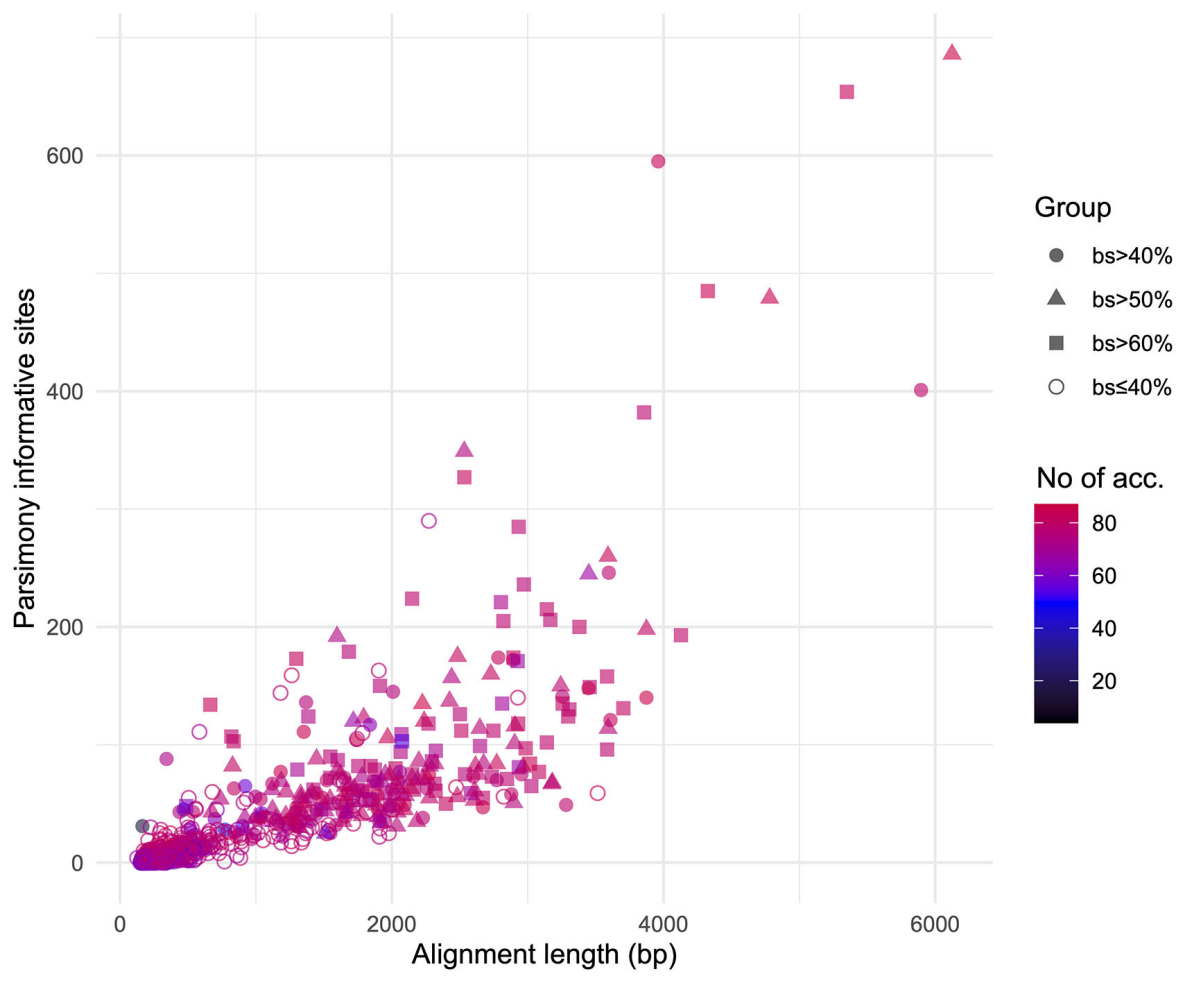
Positive relation between alignment length and parsimony informative sites for the 832 loci obtained. Different shapes identify the subsets based on the threshold values of the upper quartile of rapid bootstrap support values obtained in RAxML for each gene tree. Colors indicate the number of accessions for which each loci was obtained.

**Figure 2:**
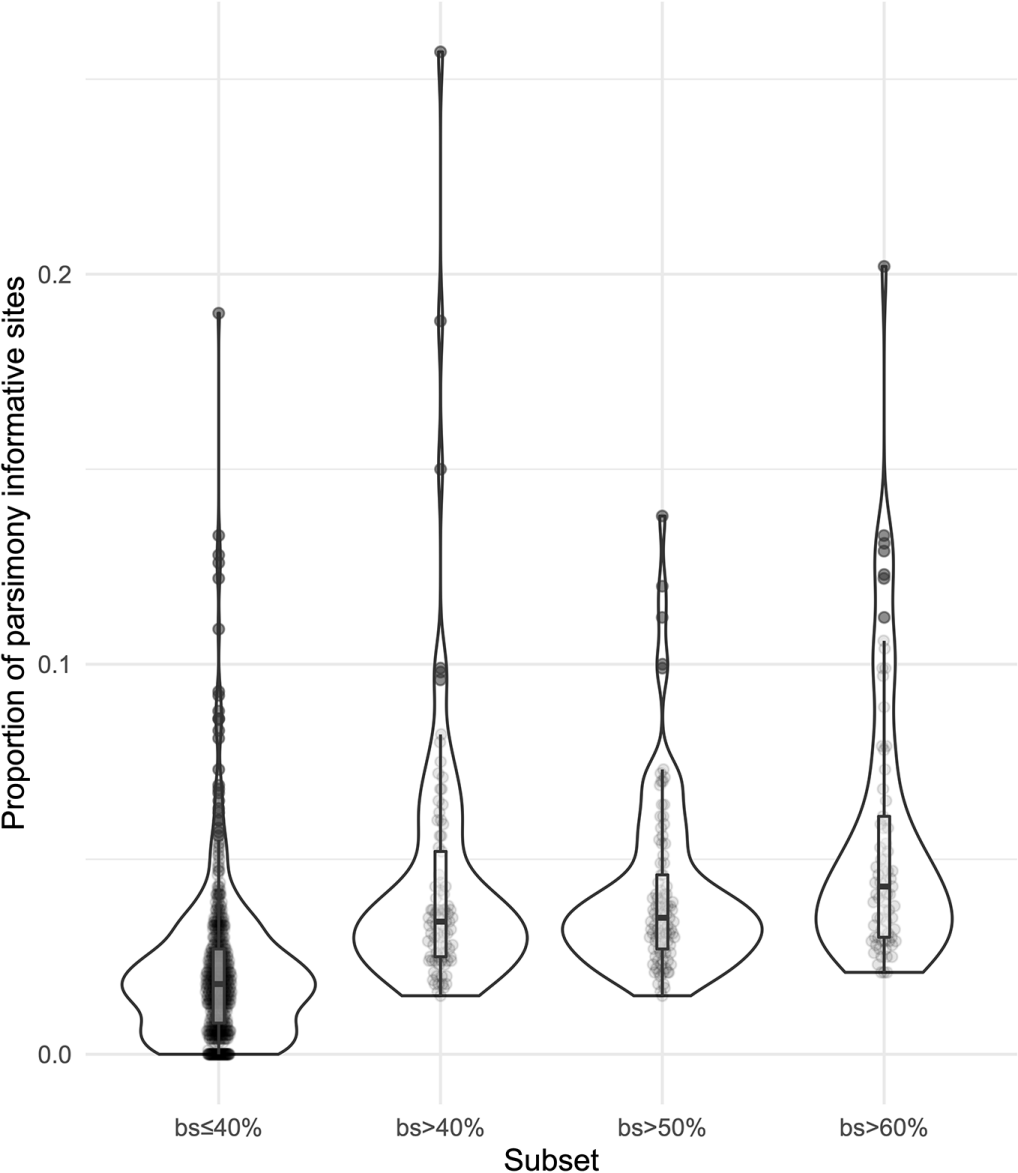
Violin plots showing the distribution of the proportion of parsimony informative sites for the subsets of loci.

We obtained on average 4.018 (SD = 2.016, Min = 0.615–Max = 9.606) million reads per accession of which 46.612% (8.889%, 27.100–64.400%) were on target and assembled on average on 1,210.600 (248.501, 162–1,355) loci per accession (**Supplementary Figure 1**). Of the target loci intended for the phylogenomic reconstruction, we obtained 1,145 aligned loci generating 881,627 aligned base pairs yielding 36,596 parsimony informative sites (PIS). 313 loci had paralogy warnings for more than 5% of the obtained sequences; the remaining 832 had 792,974 aligned base pairs with 31,462 PIS. The distribution of loci that produced gene trees with higher bootstrap support values according to the thresholds (>40, >50, and >60%) of the upper quartile of the RAxML rapid bootstrap support values is presented in **Table 1** and **Supplementary Figure 2**. The longer alignments show a tendency to have more PIS (**Figure 1**), and the proportion of PIS is significantly different among the subsets of loci (χ²[3] = 171, *p* < 0.0001; **Figure 2**). The PIS are significantly higher in the subsets of loci that yielded the gene trees with at least 40% rapid bootstrap support values in the upper quartile (bs > 40% *v.* bs ≤ 40%: Z = 8.587, adjusted *p* < 0.0001; bs > 50% *v.* bs ≤ 40%: Z = 8.566, adjusted *p* < 0.0001; bs > 60% *v.* bs ≤ 40%: Z = 11.260, adjusted *p* < 0.0001) and marginally (bs > 50% *v.* bs > 60%: Z = −3.072, adjusted *p* = 0.0128) or nonsignificantly different among them (bs > 40% *v.* bs > 50%: Z = 0.794, adjusted *p* > 0.999; bs > 40% *v.* bs > 60%: Z = −1.838, adjusted *p* = 0.396).

**Table 1:** Summary statistics of the length in base pairs and the number of parsimony informative sites (PIS) for the alignments of all the 832 loci and the subsets defined by the upper quartile of the RAxML rapid bootstrap support values of each gene tree (≤40, >40, >50, and >60%).

		Contig (bp)	PIS
All	**mean**	951.868	37.43
**n = 832**	**sd**	968.228	66.529
	**min**	126	0
	**max**	6,123	686
	**total**	791,954	31,142
≤40	**mean**	449.222	11.463
**n =568**	**sd**	411.547	21.41
	**min**	126	0
	**max**	3,515	290
	**total**	255,158	6,511
**(40,50]**	**mean**	1,684.18	69.876
**n = 89**	**sd**	957.432	77.98
	**min**	165	11
	**max**	5,895	595
	**total**	149,892	6,219
**(50–60]**	**mean**	2,031.622	87.418
**n = 98**	**sd**	847.812	90.043
	**min**	675	24
	**max**	6,123	686
	**total**	199,099	8,567
>60	**mean**	2,439.026	127.857
**n = 77**	**sd**	864.684	99.038
	**min**	487	45
	**max**	5,349	654
	**total**	187,805	9,845

### Phylogenomic Inference

**Figure 3:**
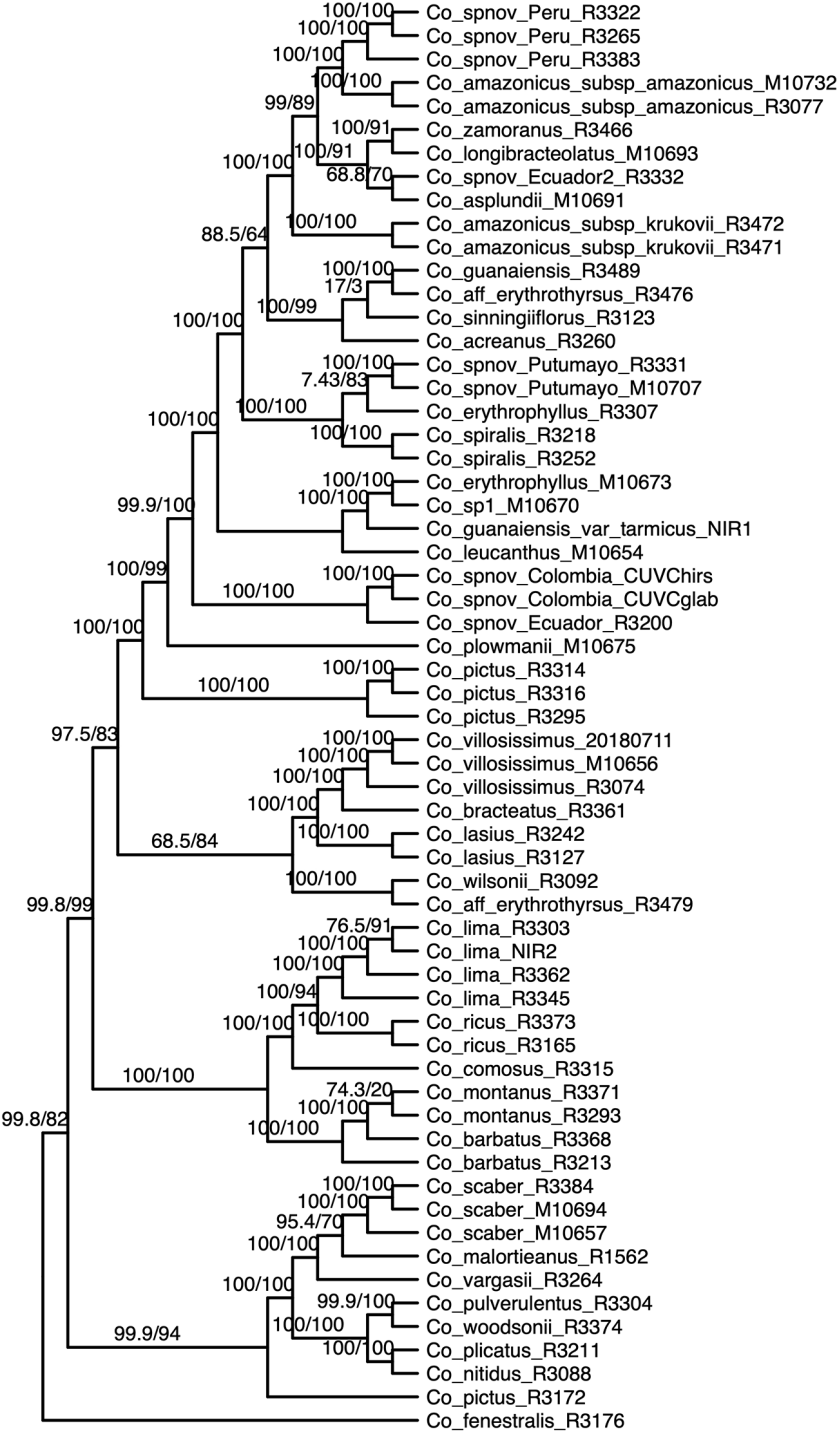
Phylogenetic reconstruction with the concatenation of 832 loci analyzed in IQ-Tree; the values above the branches are the result of the SH-aLRT (above 80 are considered strongly supported) and ultrafast bootstrap support (above 95 are considered strongly supported) showing high support values in most of the branches. Equal branch lengths were used to allow the reader to distinguish support values; branch lengths are depicted in **Figure 6**.

**Figure 4:**
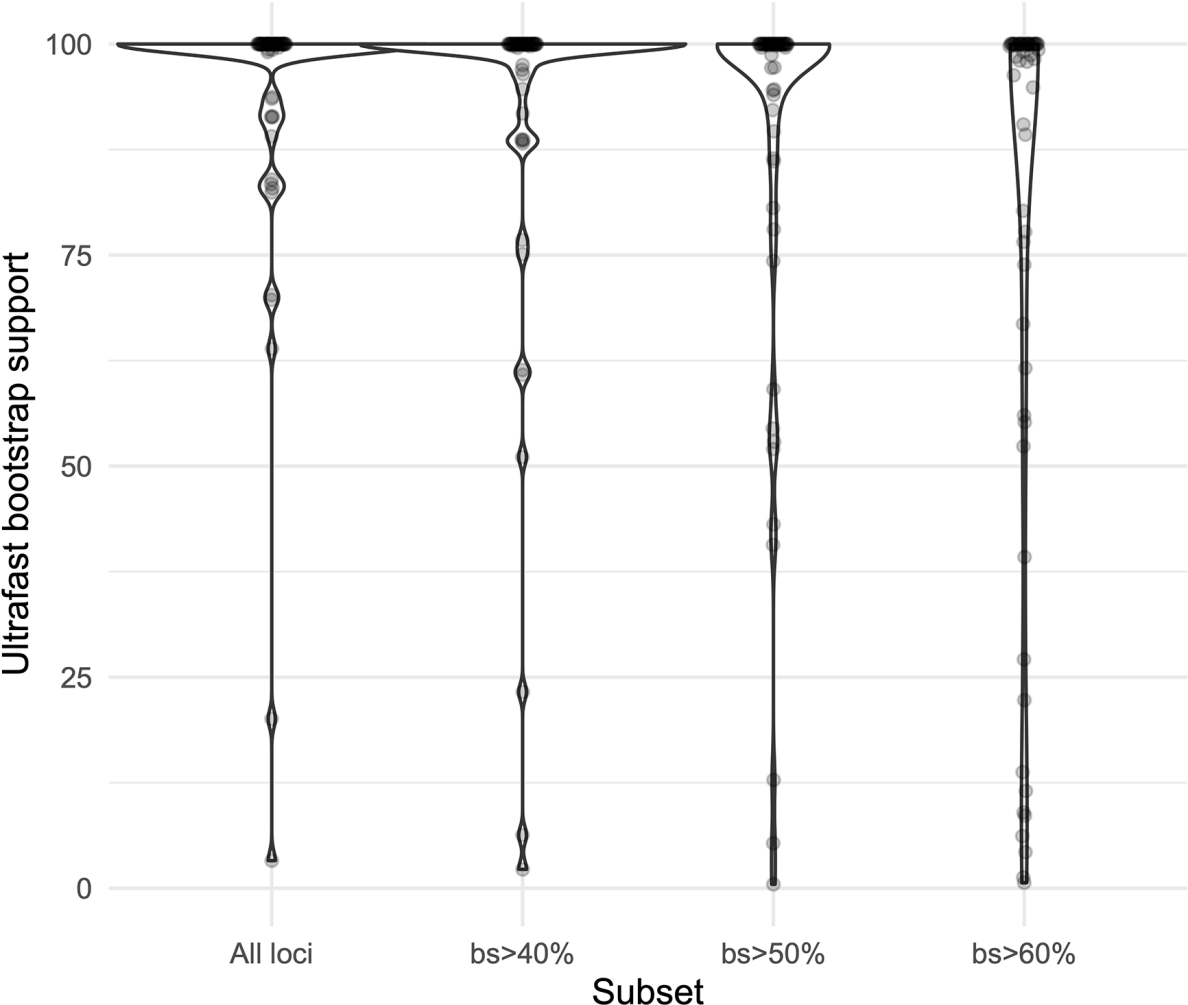
Violin plots comparing the ultrafast bootstrap support values obtained with the concatenation of all the loci and the different subsets in IQ-Tree.

We obtained high support values for most of the inferred relationships using the concatenation approach (**Figure 3**). The ultrafast bootstrap support values obtained with the different subsets of loci are significantly different (χ²[3] = 49.127, *p* < 0.0001), and the analysis with the highest levels of support is the one that includes all available loci, as compared with analyses using only loci that produced more resolved gene trees and had a higher proportion of PIS (**Figure 4**). Wilcoxon signed-rank tests showed significant differences in the comparisons of the ultrafast bootstrap support values of All loci *v.* bs > 50% (V = 501, adjusted *p* < 0.001, adjusted *r* = −0.454), All loci *v.* bs > 60% (V = 622, adjusted *p* < 0.0001, adjusted *r* = −0.540), bs > 40% *v.* bs > 50% (V = 467.5, adjusted *p* = 0.005, adjusted *r* = −0.365) and bs > 40% *v.* bs > 60% (V = 612, adjusted *p* < 0.0001, adjusted *r* = −0.518). We obtained marginal differences for bs > 40% *v.* bs > 50% (V = 515.5, adjusted *p* = 0.080, adjusted *r* = −0.228) and nonsignificant differences for All loci *v.* bs > 40% (V = 273, adjusted *p* = 0.607, adjusted *r* = −0.067). All *p* values were corrected for multiple comparisons and subsequently used to estimate the *r* values. Considering a smaller subset of the best merging schemes of substitution models for the partitions did not prevent the analysis (including all loci) to yield higher support values. The topology remains stable when the number of regions included is reduced (except for the >60% subset), but support values decay when considering fewer loci, even if those being kept are the more informative ones within the dataset (**Supplementary Figures 3A–C**). The reduction in support values is most noteworthy for the deeper nodes in the tree comprising the early diverging lineages of Neotropical *Costus*. The branch lengths of the more weakly supported backbone of the phylogeny are very short, and the values of the local posterior probability of the ASTRAL analysis are also the lowest in the tree.

**Figure 5:**
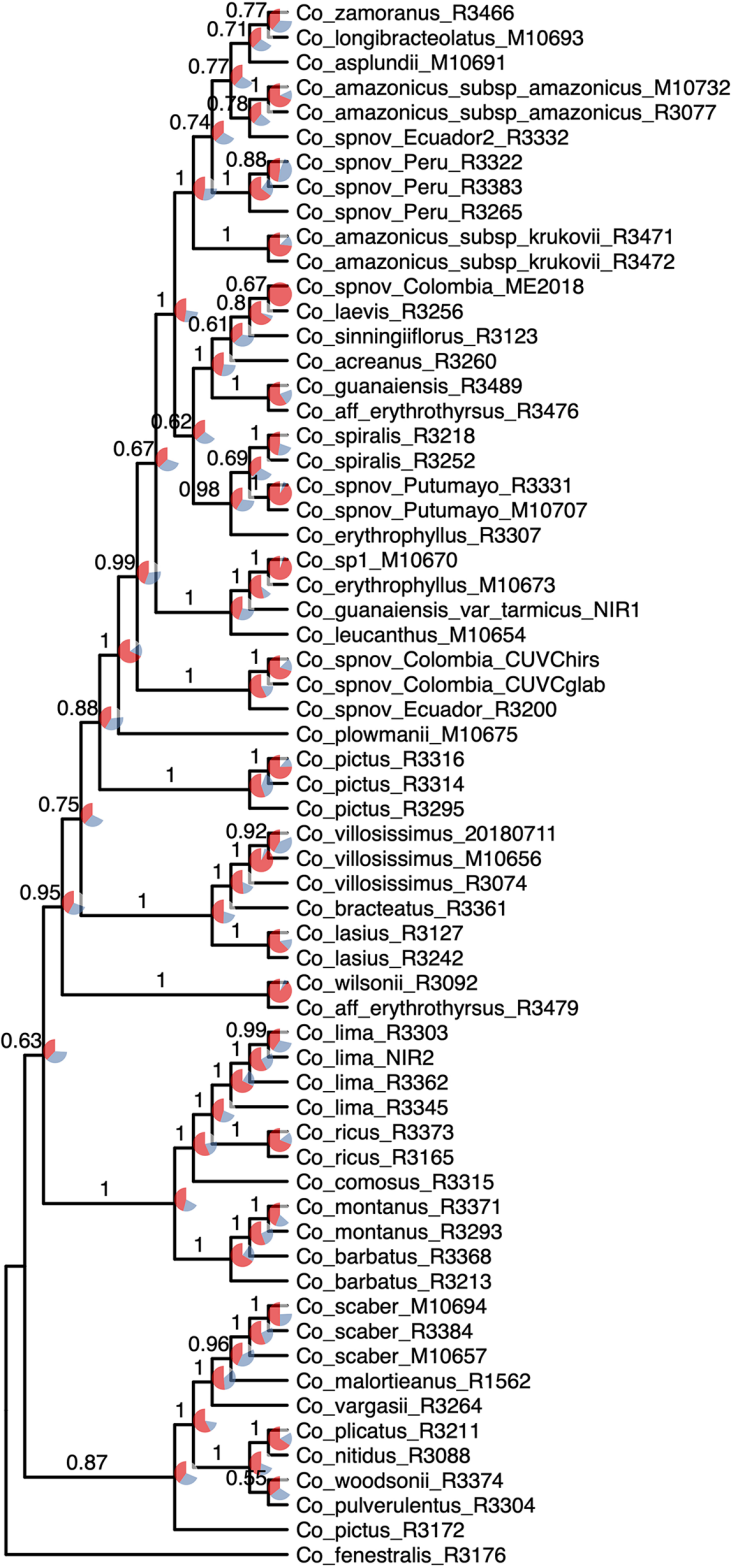
Species tree reconstruction by ASTRAL with local posterior probabilities above the branches. Pie charts illustrate the quartet scores for each node for the 832 loci, with red representing the current topology, blue the second most favored topology, and white the remaining one. Equal branch lengths were used to allow the reader to distinguish support values; branch lengths are depicted in **Figure 6**.

**Figure 6:**
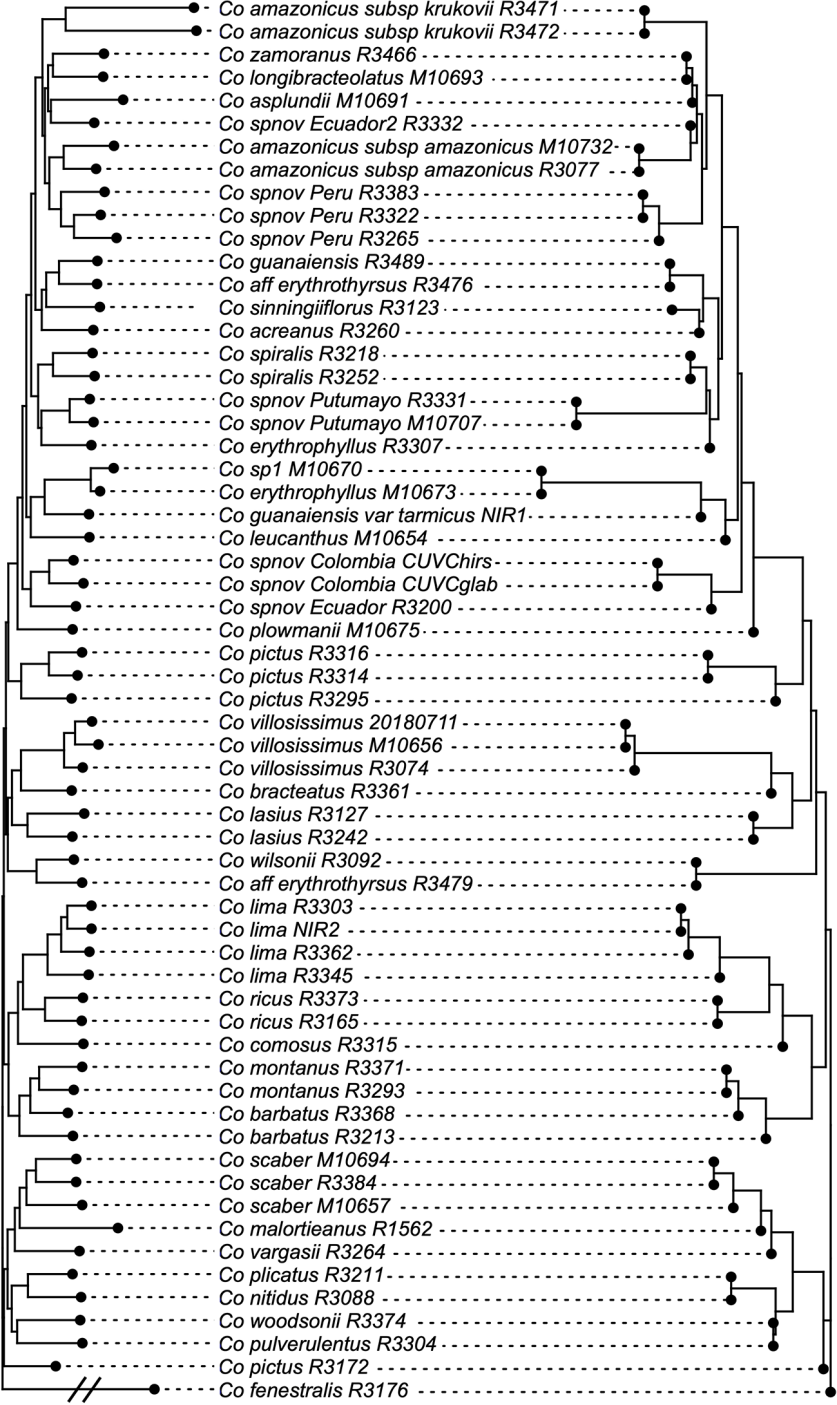
Topologies obtained with concatenation *v.* coalescent-based species tree analyses, showing just one node difference between the two. Branch lengths proportional to the number of substitutions for the IQ-Tree result and to coalescent units in the ASTRAL result.

The normalized quartet score of the topology obtained with ASTRAL is 70.778%, suggesting high levels of discordance among gene trees. The quartet scores indicate high levels of gene tree conflict in the backbone of the phylogeny; even relationships with high local posterior probabilities show that several gene trees support the alternative topologies of each quartet (**Figure 5**). Despite the high levels of conflict among gene trees, short branches in the early diverging lineages of the phylogeny and the completely different approaches used to estimate species trees, the overall topology recovered with concatenation *v.* coalescent-based species tree method is almost identical, suggesting robustness of the relationships recovered by the methodology (**Figure 6**).

Most of the species which were sampled for more than one individual are recovered as monophyletic in our resulting phylogeny, even when considering broad geographical variation (*e.g.* *Costus lima* K. Schum. with individuals sampled from Ecuador and Costa Rica, *Costus lasius* Loes. with individuals from Peru and Panama) or morphological variation (*e.g.* *Costus* sp. nov. Peru with glabrous and pubescent forms recovered as sister). Enigmatic lineages that will likely constitute new species show considerable divergence from closely related species (*e.g.* *C.* sp. nov. Colombia). In other cases, our phylogeny includes lineages that are not closely related yet are currently considered as a single species: for example, *C. amazonicus* (Loes.) subspecies *amazonicus* J.F.Macbr. and *Costus amazonicus* subspecies *krukovii* Maas, and *C. guanaiensis* varieties (incl. *Costus guanaiensis* var. *tarmicus* (Loes.) Maas). Similarly, an individual from Puerto Rico identified as *Costus pictus* D.Don is not related to the accessions of the same species from Mexico and Costa Rica. Either *Costus aff. erythrothyrsus* accessions from the Acre Region in Brazil or *Costus erythrophyllus* Loes. lineages from the foothills of the eastern and western ridges of the Colombian Andes are monophyletic clades in our results. Various accessions having intermediate morphologies that were identified as potential hybrids between species cluster with one of the species identified as possible parentals. The support values for the backbone of the phylogeny are visibly lower in the analyses that included the potential hybrids (**Supplementary Figure 4**) than the analyses where those accessions were excluded (**Figure 3**). The NeighborNet network similarly clusters potential hybrids with candidate parentals and supports the topology obtained with the other analyses (**Supplementary Figure 5**).

### Phylogenetic Comparative Methods

**Figure 7:**
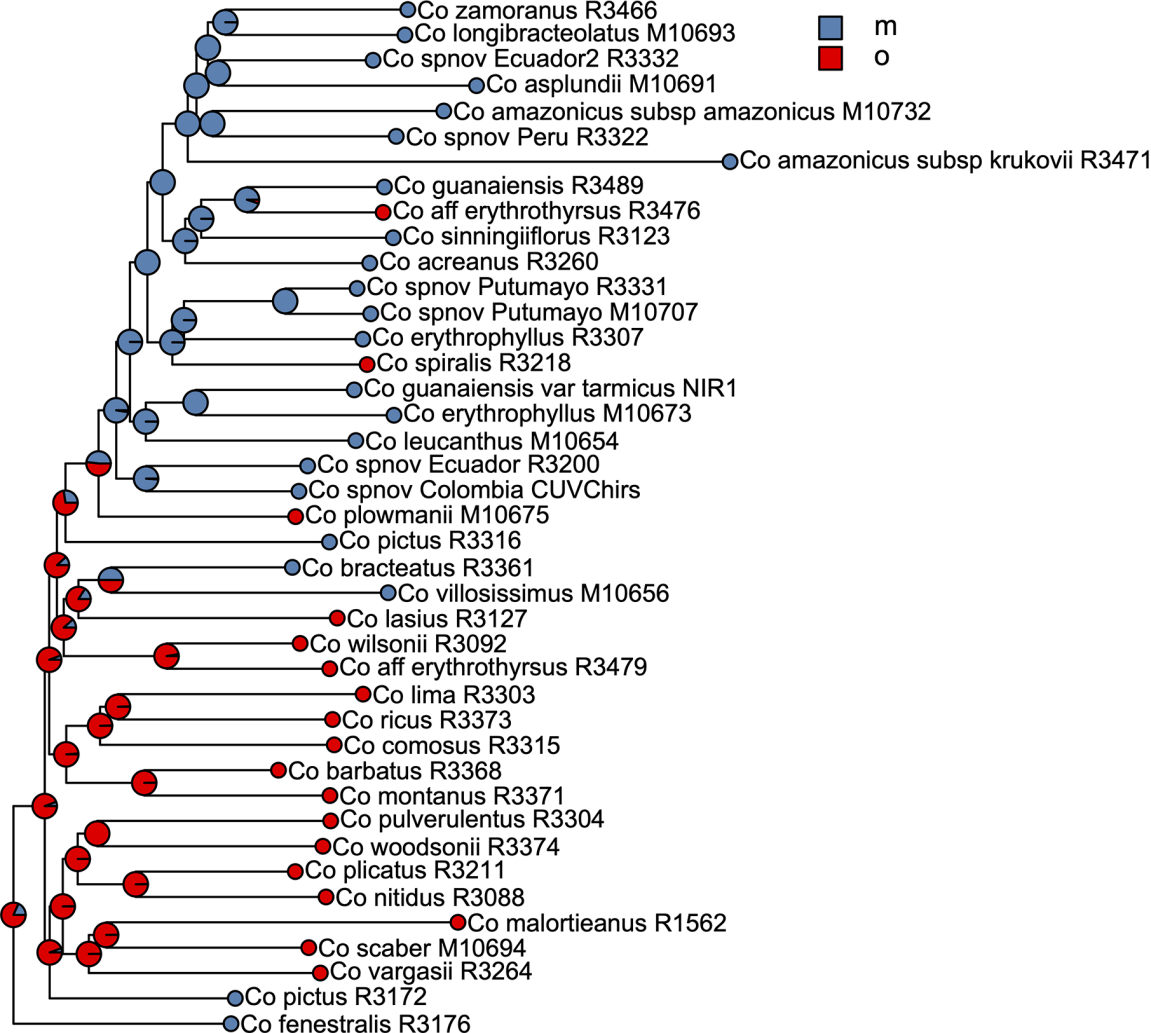
Summary of the stochastic character mapping showing multiple shifts in pollination syndromes during the history of the Neotropical *Costus*. Pie charts indicate the posterior probabilities obtained from the 1,000 stochastic mappings (m, melittophilous; o, ornithophilous).

**Figure 8:**
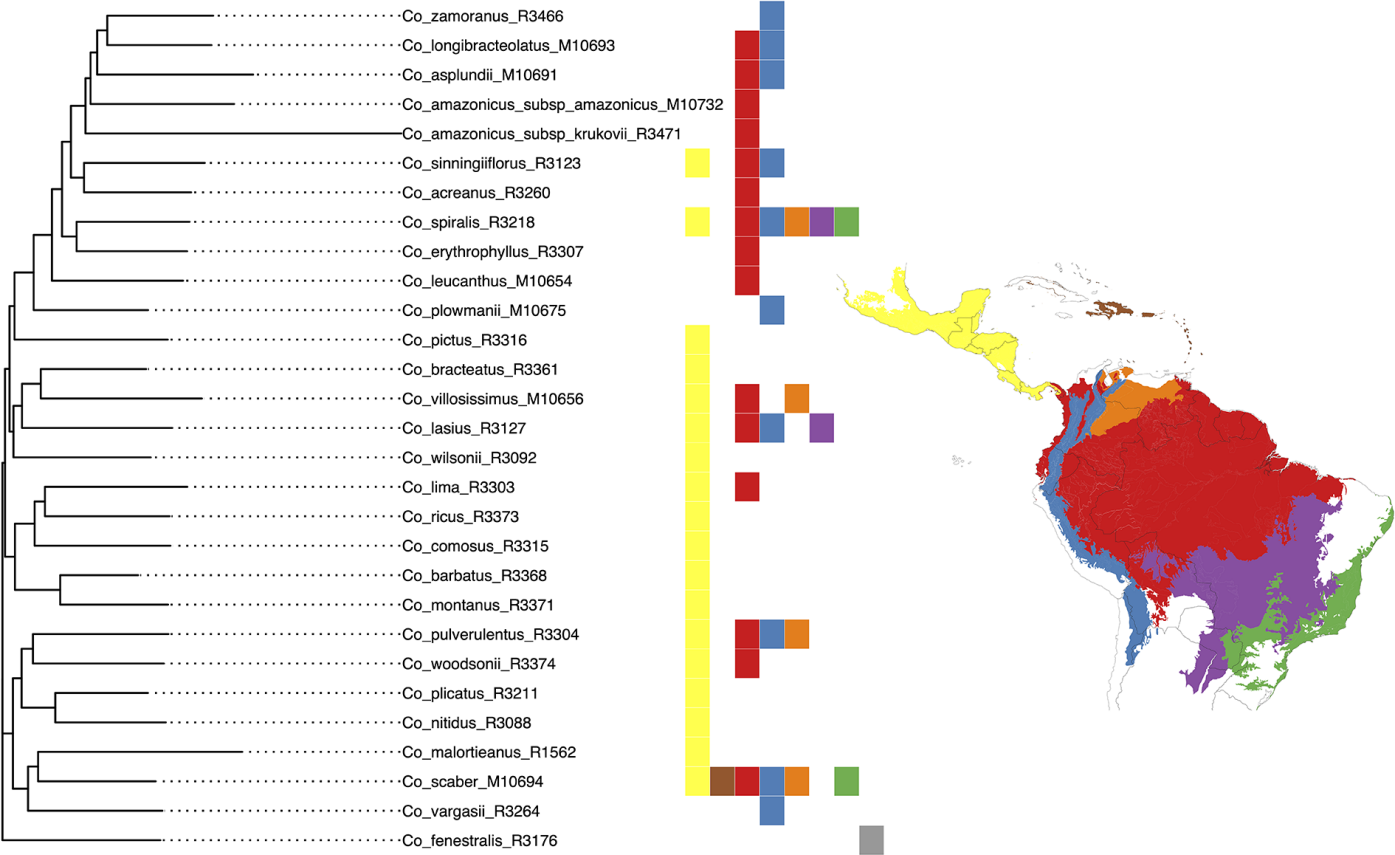
Classification of the geographical distribution of the species of *Costus* included in the analyses. The regions from north to south are 1. Mesoamerica, 2. West Indies, 3. Amazon, Interandean Valleys and Choco-Darien region, 4. Northern and Central Andes, 5. Llanos region, 6. Cerrado, and 7. Atlantic Forest.

We selected the model with equal transition rates for the shifts in pollination syndromes for the stochastic character mapping analysis because including different rates did not improve likelihood significantly (χ^2^[1] = 0.916, *p* = 0.339). Posterior probabilities indicate multiple changes in pollination syndromes during the evolutionary history of *Costus*, with shifts occurring at least four times within the Neotropical lineage. The changes involve shifts to melittophilous pollination syndromes and subsequent regains of ornithophilous flowers. Our results suggest that the most recent common ancestor of all Neotropical *Costus* species was most likely ornithophilous in form (**Figure 7**). The analysis reconstructing the evolution of the distribution range of *Costus* shows very high levels of uncertainty but also suggests a Central American origin for the genus (**Figure 8**and **Supplementary Figure 6**).

## Discussion

The custom-designed baits allowed us to gather informative loci for a good proportion of the sampled individuals. Phylogenetic signal recovered for the sampling of Neotropical *Costus* demonstrates the efficacy of using a targeted enrichment approach to estimate phylogenies in challenging plant lineages with large genomes, especially those involving rapid radiations, putative hybrids, and/or high levels of incomplete lineage sorting. The low proportion of reads recovered from the plastid genome prevented us from obtaining comparable sequences of the chloroplast and including them in the phylogenomic analysis. Our observed level of minimal capture of off-target reads has been documented in other studies (*e.g.* Villaverde et al., 2018; Forrest et al., 2019) and is perhaps attributable to highly efficient capture by our baits which were designed specifically for *Costus*. Studies that have particular interest in the plastid genome could still use similarly designed probes but increase the coverage of chloroplast regions by sequencing a mixture of captured and uncaptured libraries (Weitemier et al., 2014).

The phylogeny presented here considerably improves the resolution and support values of previous studies (Kay et al., 2005; Specht, 2006; Salzman et al., 2015; André et al., 2016), particularly providing resolution among the early branches (*i.e.* backbone) of the Neotropical *Costus* radiation. The branch lengths obtained along the backbone are relatively short, supporting the idea of a rapid radiation of the Neotropical lineages. Furthermore, normalized quartet score of the coalescent-based species tree topology indicates high levels of gene tree discordance, a result expected when incomplete lineage sorting is prevalent in the history of the group. Hybridization and the resulting introgression over the entire evolutionary history of the genus could also lead to the observed conflict in gene trees, contributing to the challenges in obtaining a well-supported phylogeny for the Neotropical *Costus*. Disentangling the influence of incomplete lineage sorting *v.* hybridization in our gene trees is not possible with the current sampling; however, more detailed sampling of various species complexes (*e.g.* *Costus comosus* (Jacq.) Roscoe; *Costus guanaiensis*) in the future could help detangle these processes particularly at the tips. Additional cases of nonmonophyletic species like *Costus amazonicus* and *Costus pictus* could be the pattern resulting from hybridization and introgression but also examples of cryptic species that require further studies on morphological and genomic evidence. Despite the challenging scenario of highly incongruent gene trees, the almost absolute concordance of the concatenation and coalescent-based species tree approach suggests that the topology obtained is stable, and the signal of the obtained loci overcomes the assumptions and caveats of the methods. The fact that the concatenation method produced the same topology as the method using a multispecies coalescent model, which explicitly accounts for incomplete lineage sorting, highlights the utility of concatenation-based methods for phylogenomic studies even in the presence of some degree of incomplete lineage sorting (Tonini et al., 2015; Streicher and Wiens, 2017). This is especially important given the high levels of gene tree incongruence present in this dataset.

Our observed decay in support values when building trees with reduced numbers of loci points to the importance of including as many loci as possible, ideally scattered across the genome (Blom et al., 2016; Bragg et al., 2018). The inclusion of more loci, even those with a lower proportion of parsimony informative sites and/or those generating poorly resolved gene trees, improved the support values of our resulting topology in concatenation analyses, particularly for the backbone where a lack of resolution has been emblematic for the Neotropical *Costus* clade. In our dataset, improvements in resolution obtained from including more loci overcome the computational restrictions in selecting schemes for merging partitions; this could be explained by the nonmutually exclusive effects of a very efficient solution for the heuristic problem (Lanfear et al., 2014) or the positive effect of gathering more phylogenetic signal when including more regions. It is important to highlight that the quartet scores indicate that relationships among the early diverging lineages of the Neotropical *Costus* show high discordance among gene trees. Even for some branches with relatively high local posterior probabilities, the quartet scores for the backbone of the current topology are low, suggesting that many loci support each of the alternative topologies in the quartets.

The ancestral area reconstruction shows very high uncertainty, probably due to the very short branches along the backbone of the phylogeny. Overall, our results agree with Salzman et al. (2015) in suggesting a Central American origin of Neotropical *Costus* species. Our results for the evolution of pollination syndrome morphology also agree with previous studies, indicating multiple shifts between bee- and bird-associated morphology occurring throughout the history of the genus. Results from stochastic character mapping suggest that the most recent common ancestor of all New World *Costus* most likely had a bird-pollinated form. Because most of the African species are insect pollinated (Maas-van de Kamer et al., 2016) and have either a melittophilous or generalist pollination form, our results point to an early appearance of the ornithophilous pollination syndrome in the ancestors of the Neotropical *Costus*. Furthermore, we confirm the reversal to a melittophilous form from ornithophilous morphology to have taken place at least twice and up to four times given our sampling (**Figure 7**). Interestingly, we also find evidence of regains of the bird pollinated flowers with high support in *Costus aff. erythrothyrsus* Loes. and *Costus spiralis* (Jacq.) Roscoe and with high uncertainty in *Costus plowmanii* Maas. These three lineages can be found at mid elevations (*c.* 1,000 m.), and the interaction with the highly diverse community of Neotropical montane birds (Quintero and Jetz, 2018) could have triggered those changes in morphology (Salzman et al., 2015). Establishing a temporal framework for these events will allow us to test the relationship of the shifts in pollination syndrome with the dramatic changes in the landscape that took place in the Neotropical region during the last 20 million years and elucidate the mechanisms that led to the high species richness in this clade perhaps resulting from an interaction between biotic and abiotic factors (Antonelli and Sanmartín, 2011). It is important to highlight that while including more species in our phylogeny and character mapping could change the specific results, overall agreement with the previous studies in the group suggests that the pattern of repeated shifts in overall floral form associated with pollinators is robust (Salzman et al., 2015; André et al., 2016).

Our phylogeny provides a guide for resolving problematic taxonomic hypothesis by testing and confirming monophyly when considering geographical and morphological variations within the described species. It also helps place enigmatic and undescribed lineages by comparing them carefully with their closest relatives. Some widely distributed and variable species are likely to be split into separate taxonomic units, thereby adjusting the taxonomy to accurately reflect evolutionary, morphological, and geographical variation. It is clear that diversity in the genus is underestimated by the current taxonomy and urges for an updated taxonomic revision. The potential to apply the baits described in this study to obtain similar datasets for a comprehensive sampling of all spiral gingers, including African taxa and the diversity only available as herbarium specimens, will allow us to test the hypothesis regarding the genetic mechanisms underlying the evolution of floral form and the recurrent changes in floral characters shown by closely related ornithophilous and melittophilous species. Finally, hybridization and introgression are likely to have been prevalent in the diversification of *Costus* in the Neotropics; a genome-wide dataset including comprehensive sampling of the diversity within the genus will allow us to test the prevalence and the directionality of hybridization events to better understand the role of reticulate evolution in the origin and diversification of the Neotropical spiral gingers.

## Data Availability Statement

The datasets and scripts generated for this study can be found in the Open Science Framework https://osf.io/fkj2x and raw reads in NCBI BioProject http://www.ncbi.nlm.nih.gov/bioproject/639561.

## Author Contributions

CDS conceived of the project and gathered the preliminary data. PM, HM-K, and DS provided cultivated and field-collected materials of otherwise impossible-to-get taxa representing documented morphologic and biogeographic variation. EV collected data, analyzed data, and wrote the manuscript. CDS, DS, PM, HM-K, and EV contributed to tissue collection, sampling and database management. MP-V and CG collected data and contributed to database management. CS collected and analyzed data. JL helped with analyses. All authors contributed to the article and approved the submitted version.

## Funding

Research in this paper was supported by funds from Cornell University’s College of Agriculture and Life Sciences and the School of Integrative Plant Science. No federal support was used for this research.

## Conflict of Interest

The authors declare that the research was conducted in the absence of any commercial or financial relationships that could be construed as a potential conflict of interest.
