# Supplementary material for: Unraveling the Spiraling Radiation: A Phylogenomic Analysis of Neotropical Costus L
Source: Front Plant Sci. 2020 Aug 14;11:1195. doi: 10.3389/fpls.2020.01195 (PMC7456938; doi:10.3389/fpls.2020.01195)
Supplement: Supplementary file 1 [file DataSheet_1.pdf]

Supplementary Material

1 Supplementary Data

Stubby Adapter sequences:

5'-/5Phos/GATCGGAAGAGCACACGTCTGAACTCCAGTC-3'

5'-ACACTCTTTCCCTACACGACGCTCTTCCGATC*T-3'

```
5'-    G A T C G G A A G A G C A C A C G T C T G A A C T C C A G T C   -3'
       | | | | | | | | | | | |                 |             | | |
3'-  T C T A G C C T T C T C G C A G C A C A T C C C T T T C T C A C A -3'
```

2 Supplementary Figures and Tables

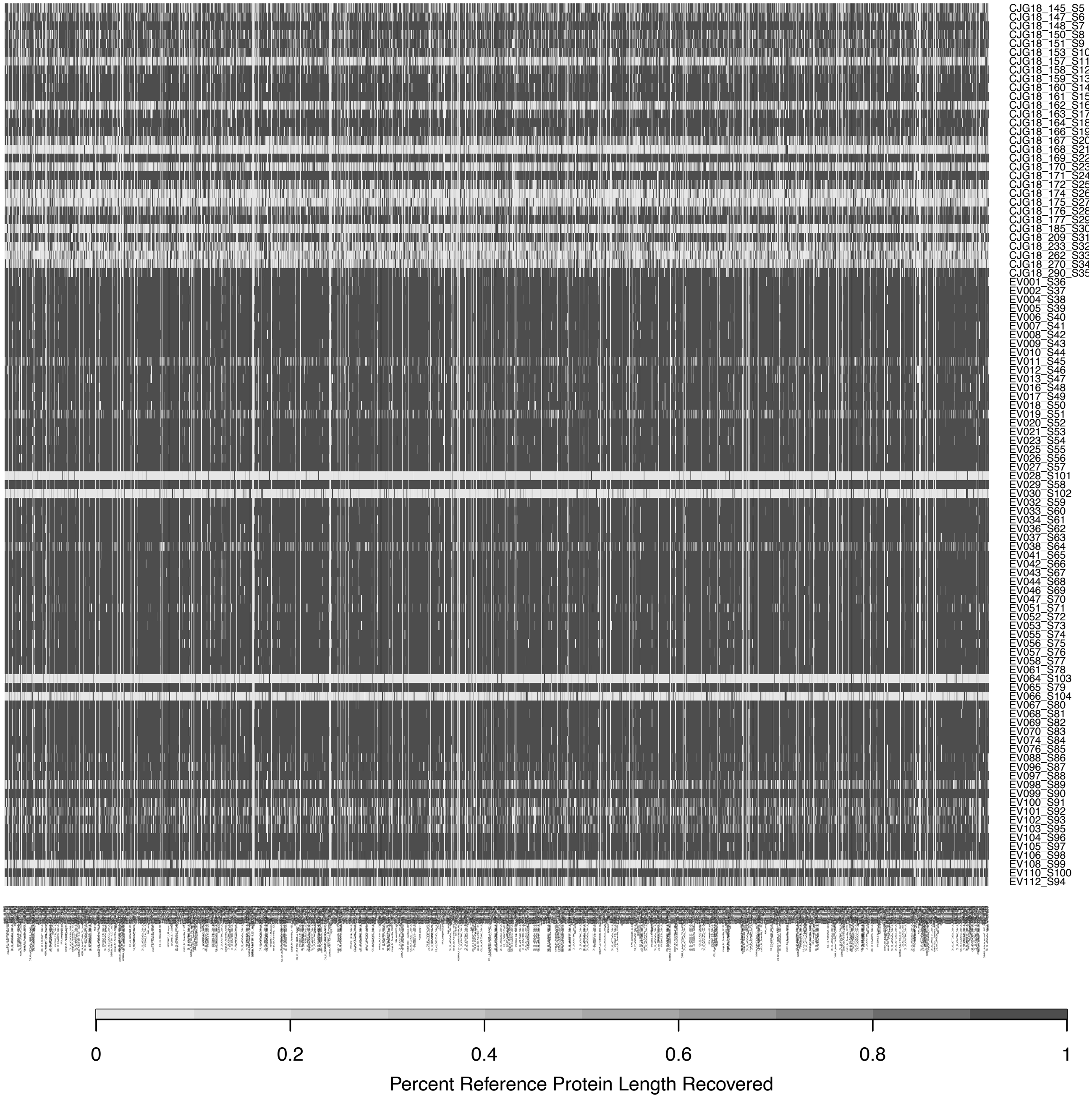

Supplementary Figure 1. Heatmap showing the percent of the target recovered of each locus (columns) for each accession (rows) to illustrate the contigs recovered per accession.

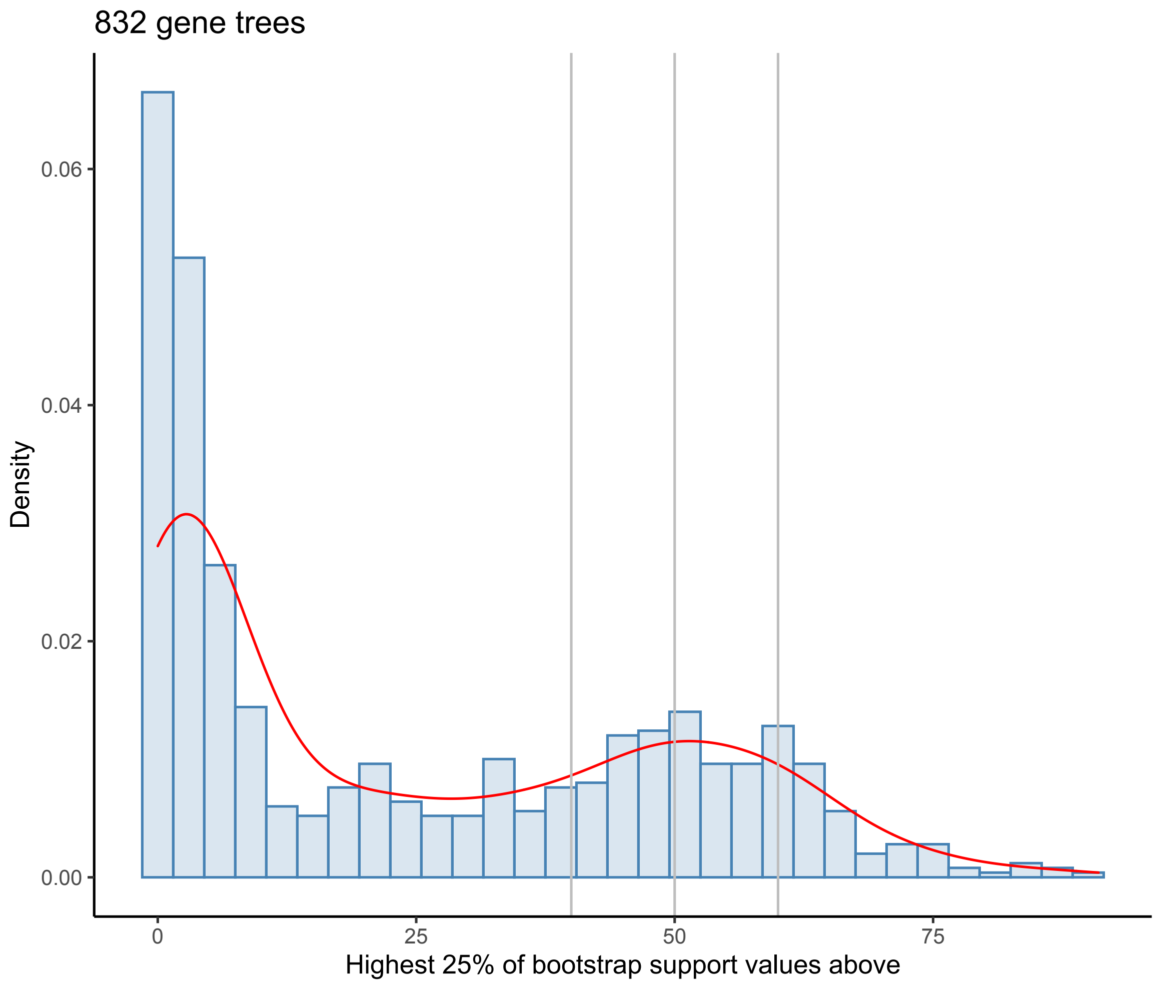

Supplementary Figure 2. Distribution of the upper quartile of the RAxML rapid bootstrap support values of the gene trees. The thresholds we used for the subsets of the 832 obtained loci are shown as grey lines.

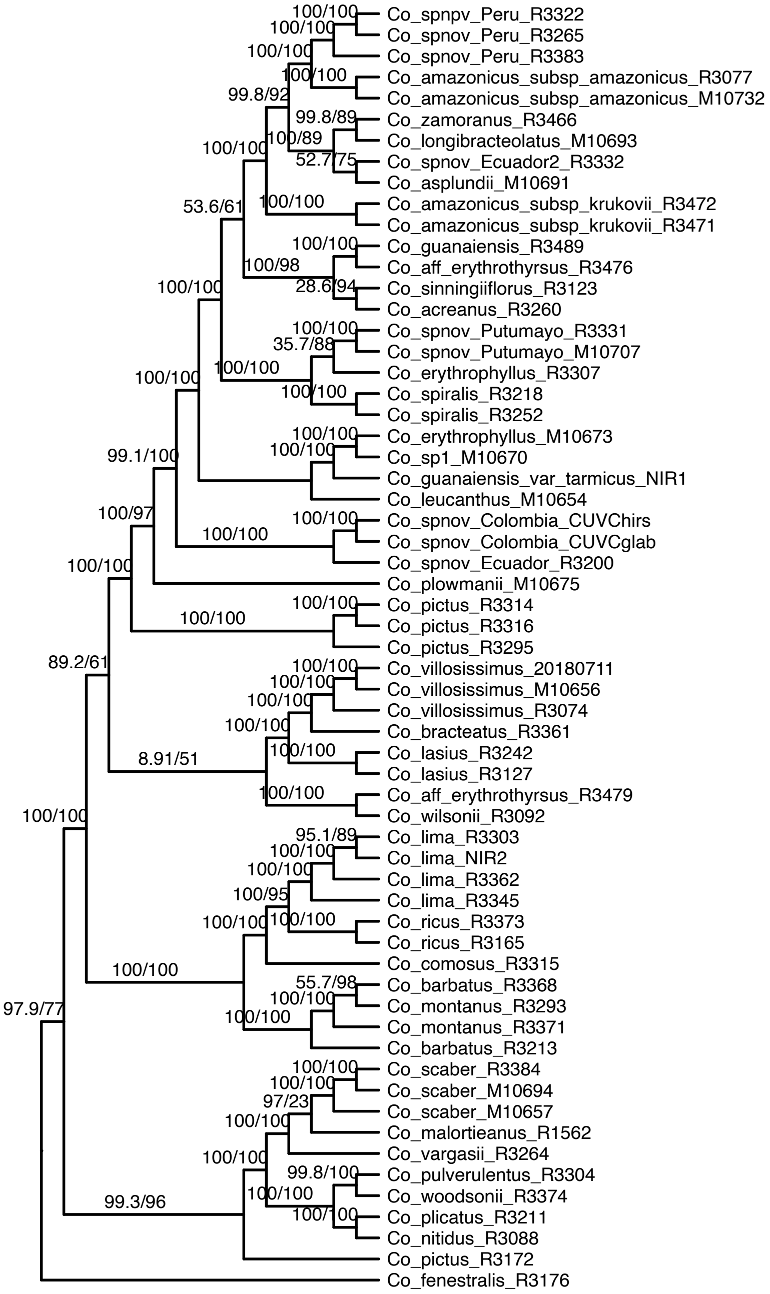

Supplementary Figure 3a. Phylogenetic reconstructions with the concatenation of **(a)** 264, **(b)** 175, and **(c)** 77 loci (of the >40%, >50 and >60% subsets, respectively) analyzed in IQ-Tree, the values above the branches are the result SH-aLRT (above 80 are considered strongly supported) and of the ultrafast bootstrap support (above 95 are considered strongly supported). Equal branch lengths were used to allow the reader to distinguish support values.

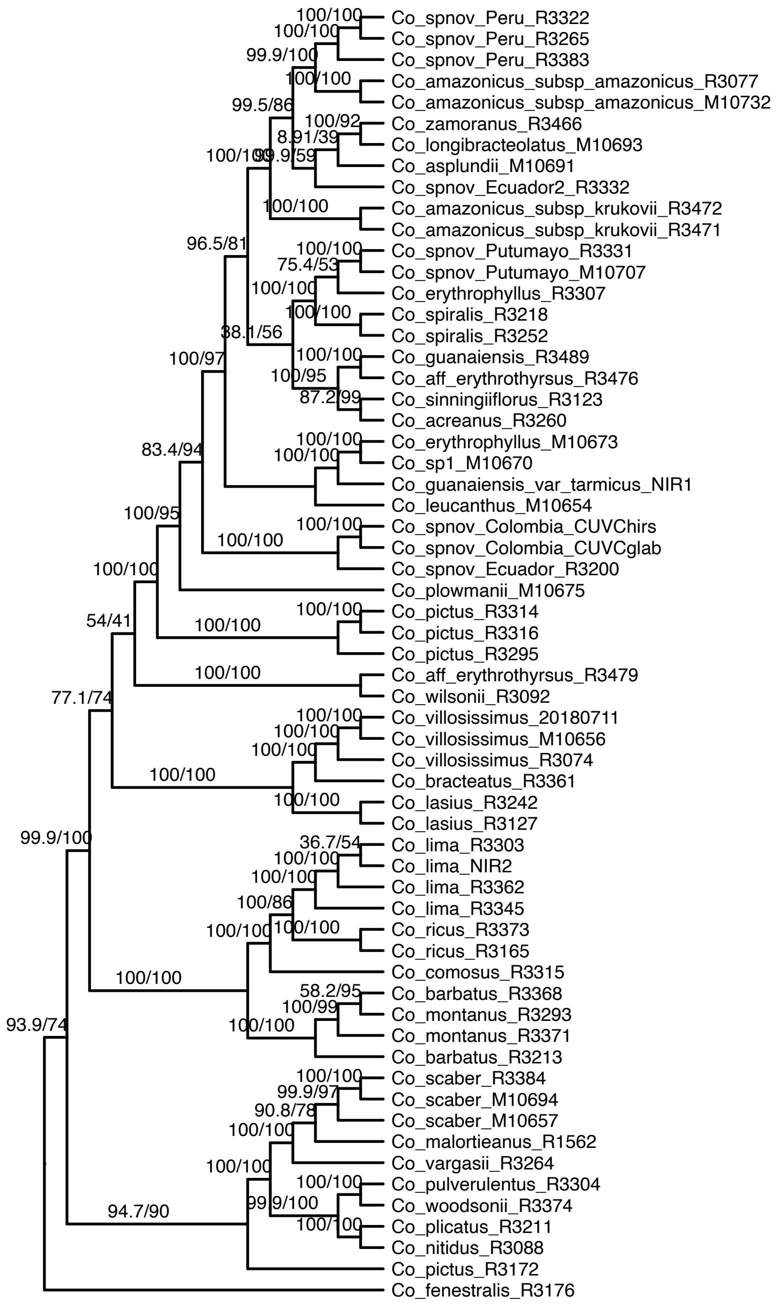

Supplementary Figure 3b. Phylogenetic reconstructions with the concatenation of **(a)** 264, **(b)** 175, and **(c)** 77 loci (of the >40%, >50 and >60% subsets, respectively) analyzed in IQ-Tree, the values above the branches are the result SH-aLRT (above 80 are considered strongly supported) and of the ultrafast bootstrap support (above 95 are considered strongly supported). Equal branch lengths were used to allow the reader to distinguish support values.

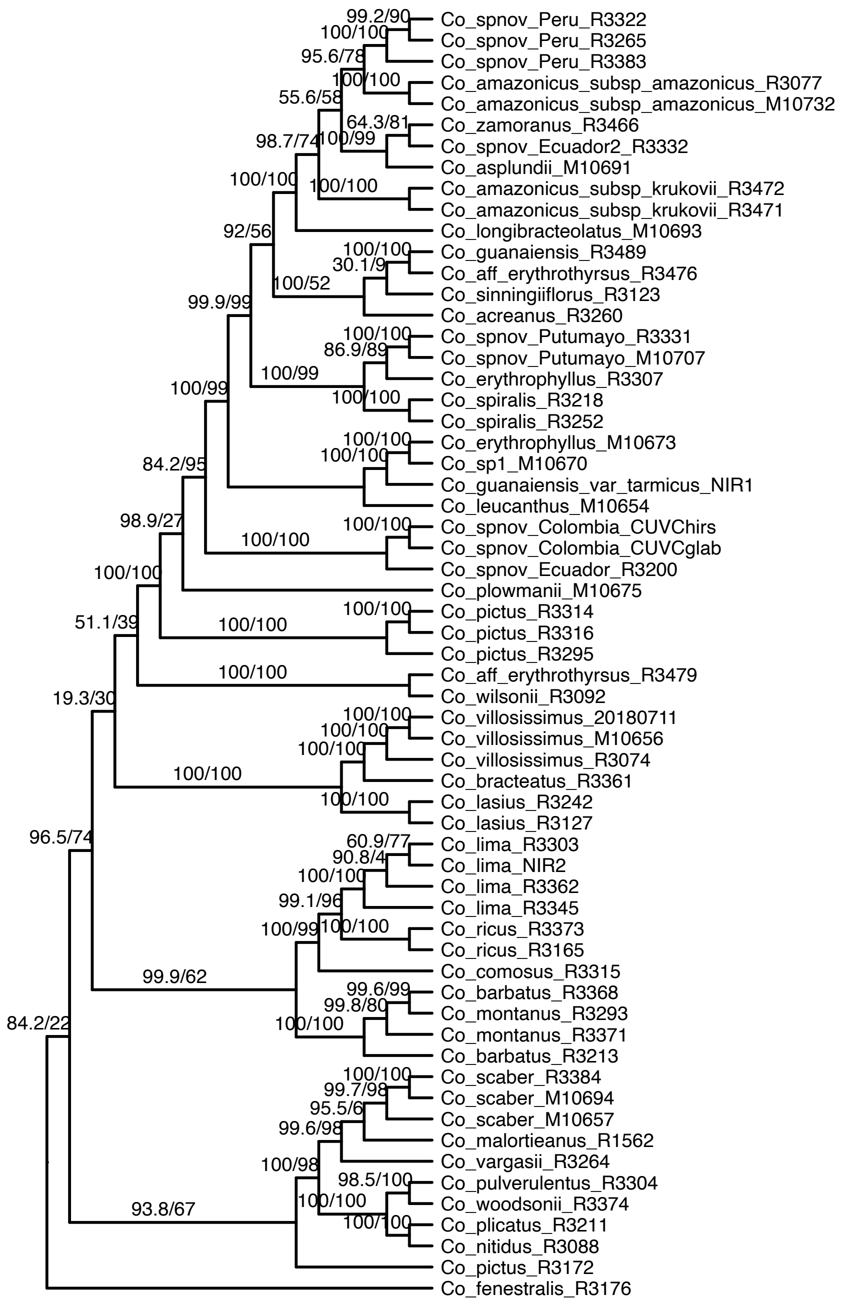

Supplementary Figure 3c. Phylogenetic reconstructions with the concatenation of **(a)** 264, **(b)** 175, and **(c)** 77 loci (of the >40%, >50 and >60% subsets, respectively) analyzed in IQ-Tree, the values above the branches are the result SH-aLRT (above 80 are considered strongly supported) and of the ultrafast bootstrap support (above 95 are considered strongly supported). Equal branch lengths were used to allow the reader to distinguish support values.

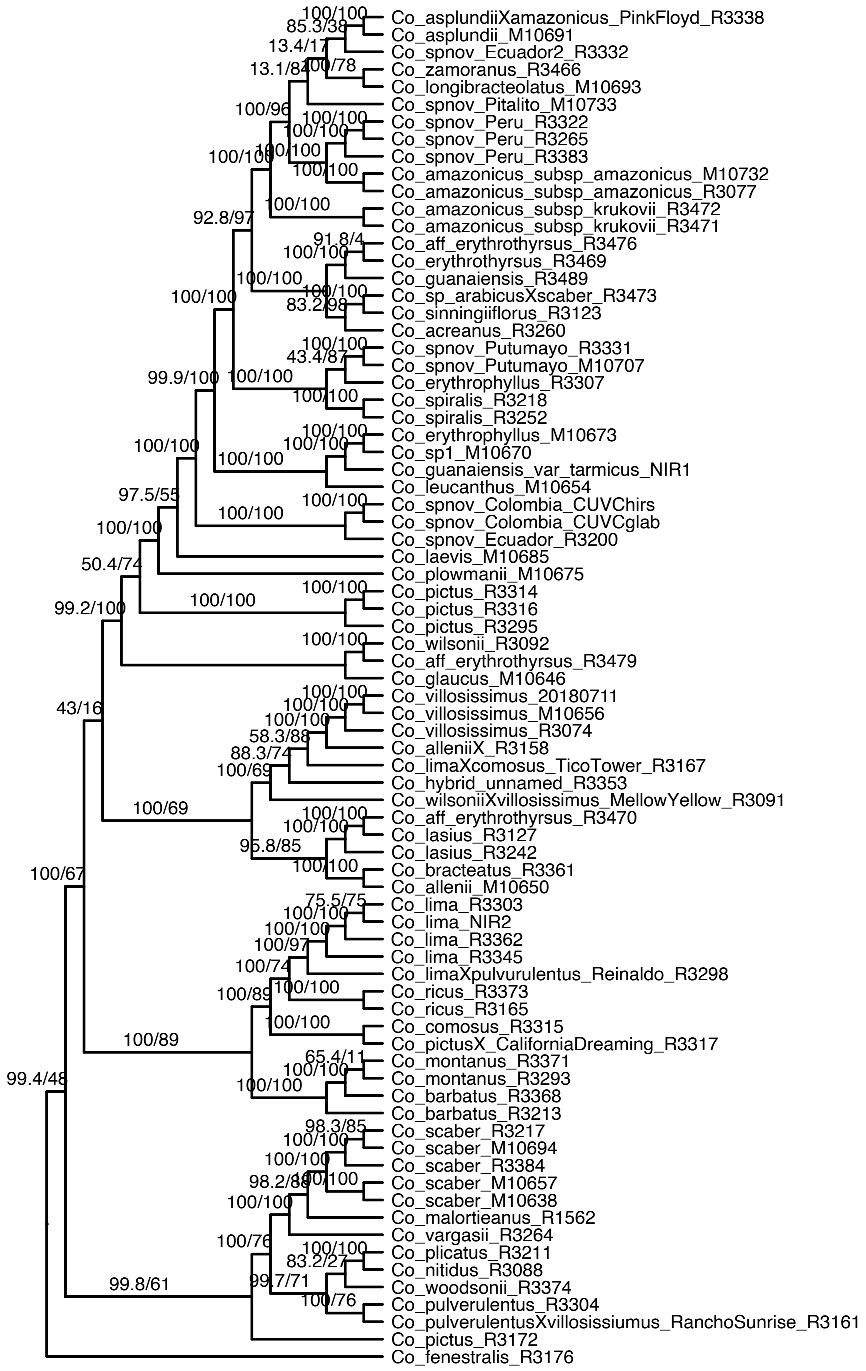

Supplementary Figure 4. Phylogeny estimated with the concatenated 832 loci in IQ-Tree including the potential hybrid accessions that can be recognized by including X in the label. The values above the branches are the result of the SH-aLRT (above 80 are considered strongly supported) and ultrafast bootstrap support (above 95 are considered strongly supported). Equal branch lengths were used to allow the reader to distinguish support values.

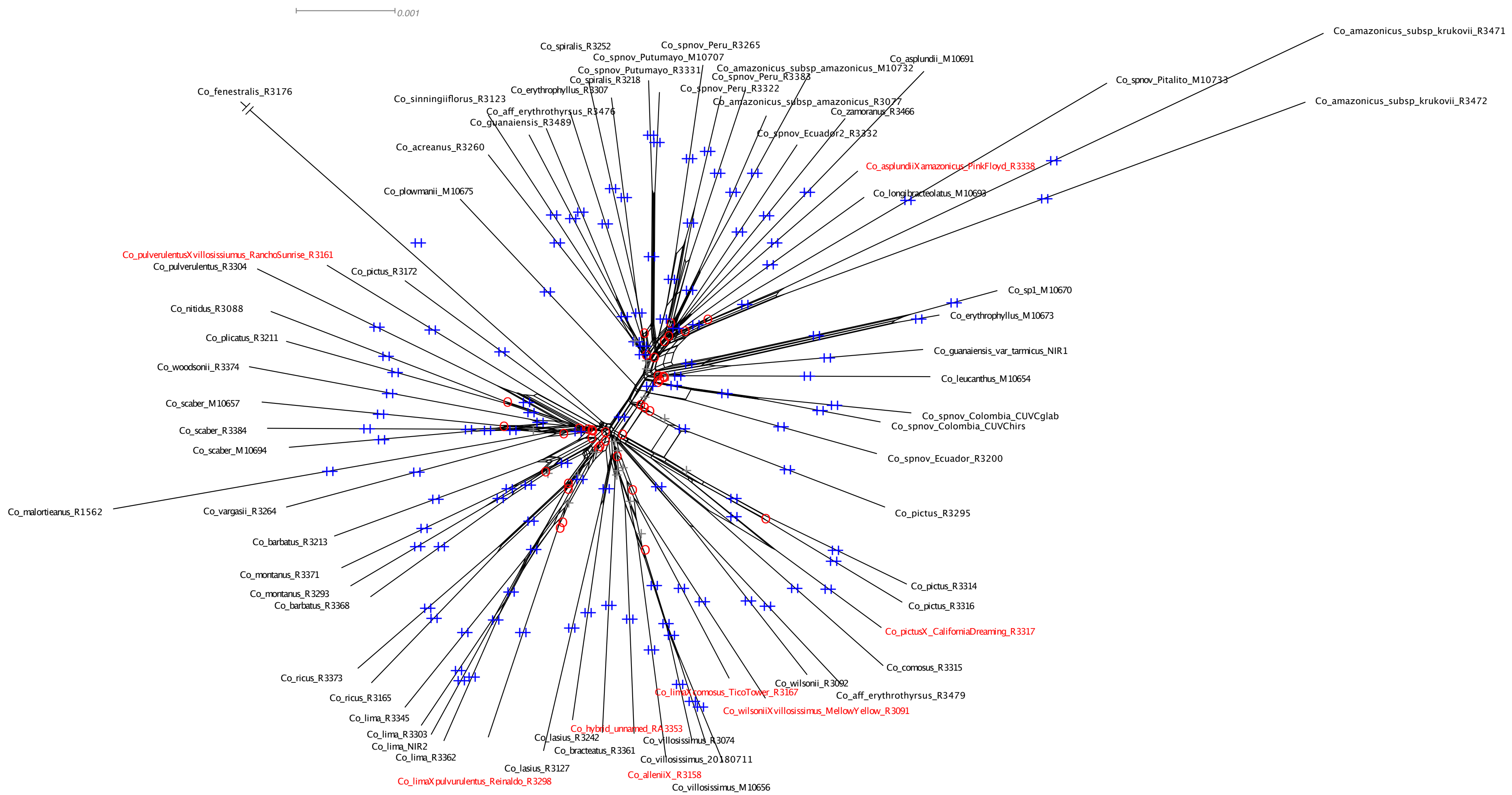

0.001
Co_amazonicus_subsp_krukovii_R3471
Co_spiralis_R3252
Co_spnov_Peru_R3265
Co_spnov_Putumayo_M10707
Co_spnov_Putumayo_R3331
Co_amazonicus_subsp_amazonicus_M10732
Co_asplundii_M10691
Co_spiralis_R3218
Co_spnov_Peru_R3383
Co_erythrophyllus_R3307
Co_spnov_Peru_R3322
Co_fenestralis_R3176
Co_sinningiiflorus_R3123
Co_amazonicus_subsp_amazonicus_R3077
Co_aff_erythrothyrsus_R3476
Co_zamoranus_R3466
Co_guanaiensis_R3489
Co_spnov_Pitalito_M10733
Co_amazonicus_subsp_krukovii_R3472
Co_spnov_Ecuador2_R3332
Co_acreanus_R3260
Co_asplundiiXamazonicus_PinkFloyd_R3338
Co_plowmanii_M10675
Co_longibracteolatus_M10693
Co_pulverulentusXvillosissiumus_RanchoSunrise_R3161
Co_pulverulentus_R3304
Co_pictus_R3172
Co_sp1_M10670
Co_nitidus_R3088
Co_erythrophyllus_M10673
Co_plicatus_R3211
Co_guanaiensis_var_tarmicus_NIR1
Co_woodsonii_R3374
Co_leucanthus_M10654
Co_scaber_M10657
Co_spnov_Colombia_CUVCglab
Co_spnov_Colombia_CUVChirs
Co_scaber_R3384
Co_scaber_M10694
Co_spnov_Ecuador_R3200
Co_malortieanus_R1562
Co_vargasii_R3264
Co_pictus_R3295
Co_barbatus_R3213
Co_montanus_R3371
Co_montanus_R3293
Co_pictus_R3314
Co_barbatus_R3368
Co_pictus_R3316
Co_pictusX_CaliforniaDreaming_R3317
Co_comosus_R3315
Co_ricus_R3373
Co_wilsonii_R3092
Co_limaXcomosus_TicoTower_R3167
Co_aff_erythrothyrsus_R3479
Co_ricus_R3165
Co_wilsoniiXvillosissimus_MellowYellow_R3091
Co_lima_R3345
Co_hybrid_unnamed_RA3353
Co_lima_R3303
Co_lasius_R3242
Co_villosissimus_R3074
Co_lima_NIR2
Co_bracteatus_R3361
Co_lima_R3362
Co_villosissimus_20180711
Co_lasius_R3127
Co_alleniiX_R3158
Co_limaXpulvurulentus_Reinaldo_R3298
Co_villosissimus_M10656

Supplementary Figure 5. Network obtained with NeighborNet in SplitsTree for the concatenated 832 loci. Potential hybrids are highlighted in red. Edges with bootstrap support values between 50% and 75% are labeled with a red 'O', the edges with bootstrap support values between 75% and 90% are indicated with a grey '+' and the edges with bootstrap support values above 90% with a blue '++'.

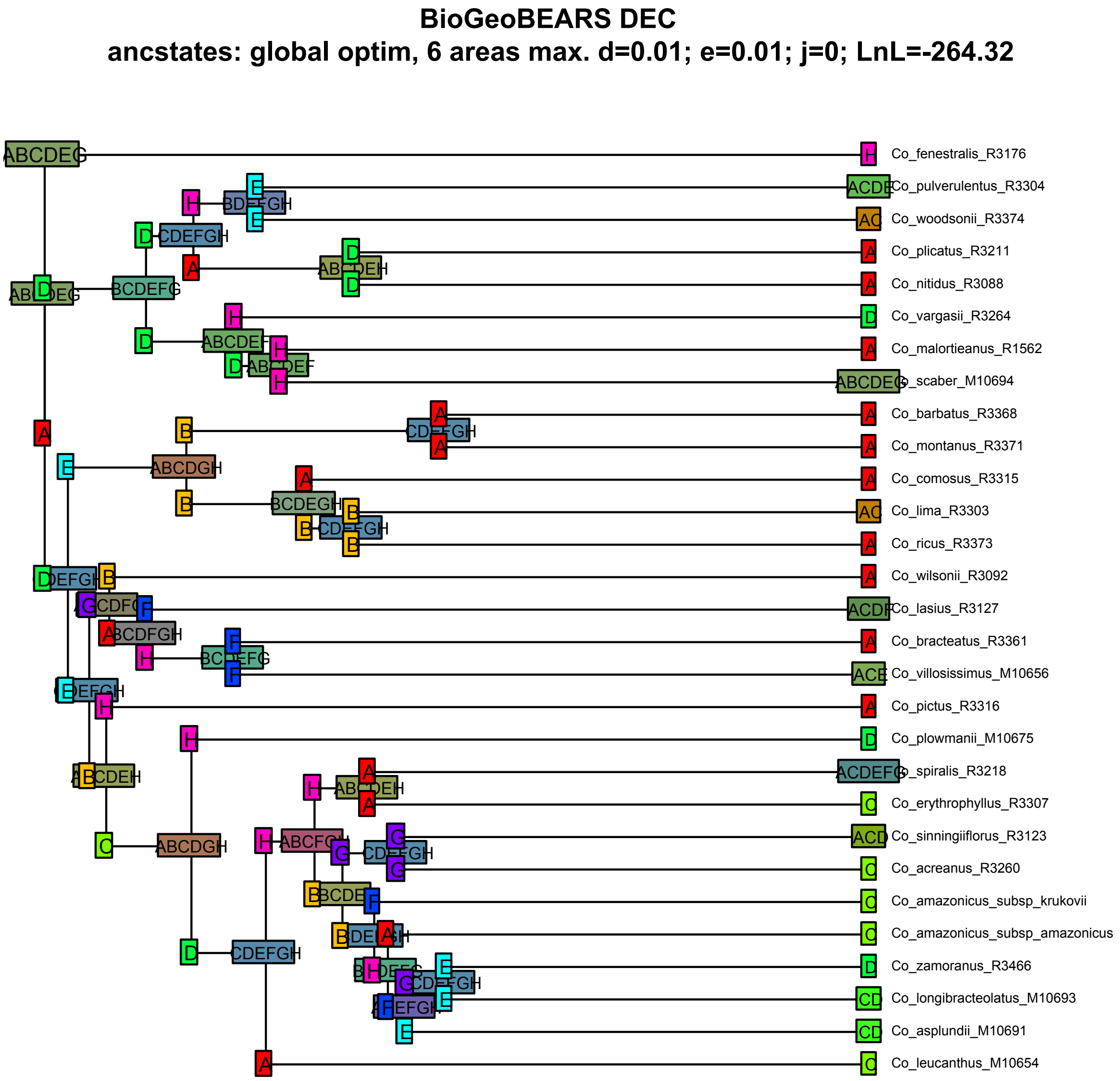

Supplementary Figure 6a. The single most-probable ancestral range model obtained with DEC in BioGeoBEARS suggesting Mesoamerica as the distribution area of most of the ancestral lineages of Neotropical *Costus*. Areas are coded as single letters as A=Mesoamerica, B= West Indies, C= Amazon, Interandean Valleys and Choco-Darien region, D= Northern and Central Andes, E = Llanos region, F= Cerrado, G = Atlantic Forest and H=Africa.

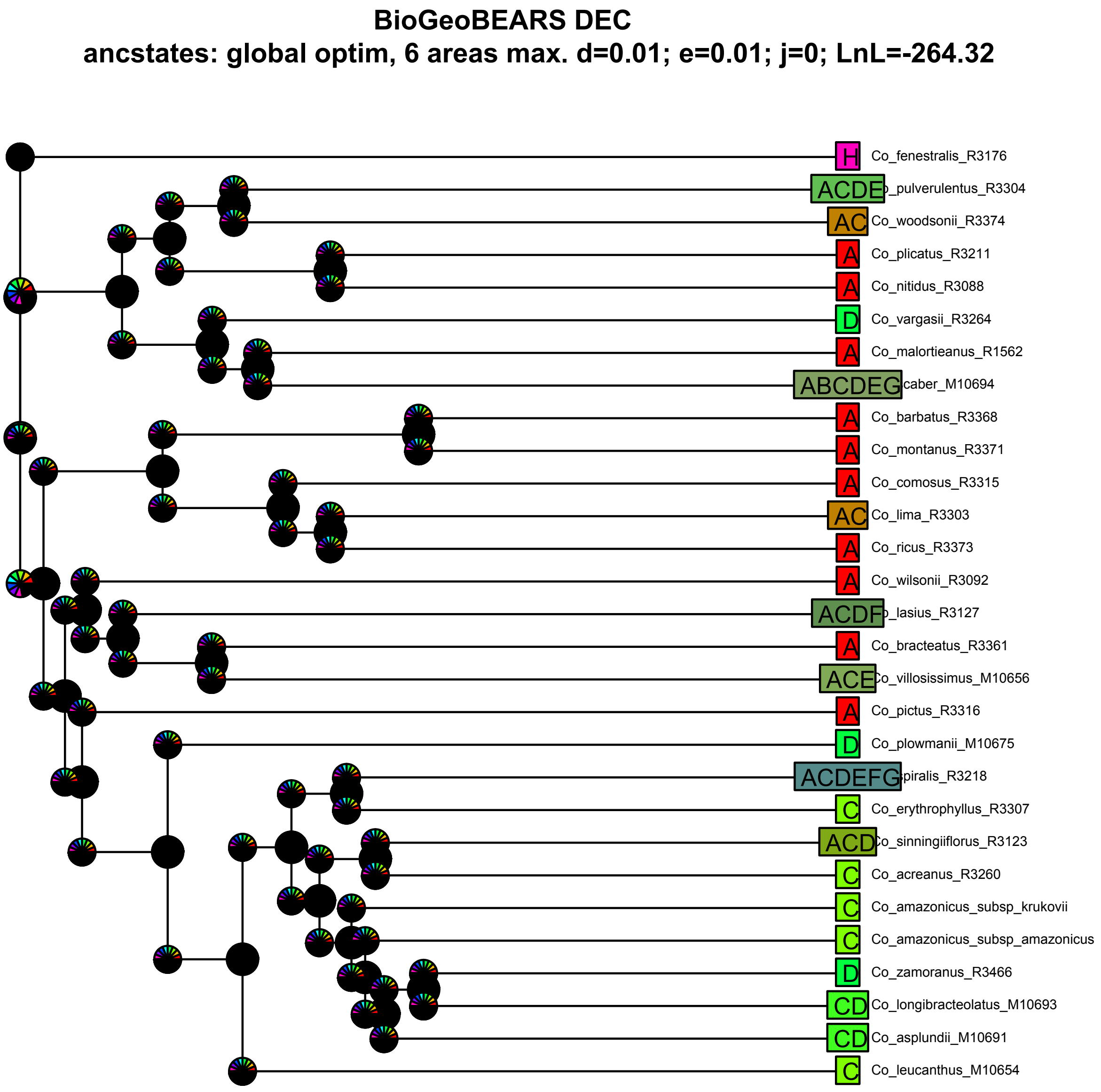

Supplementary Figure 6b. Pie charts show the relative probability of all possible geographic ranges when fitting the DEC model to Neotropical *Costus* species showing very high levels of uncertainty. Areas are coded as single letters as A=Mesoamerica, B= West Indies, C= Amazon, Interandean Valleys and Choco-Darien region, D= Northern and Central Andes, E = Llanos region, F= Cerrado, G = Atlantic Forest and H=Africa.

Supplementary Table 1. ID in the figures, collector number, species, locality and collection were the samples were retrieved. Type of tissue refers to fresh material dried in silica gel (F) and herbarium specimens (H). Herbarium acronyms followed by an L (MEDEL-L) refers to living collections associated to the herbarium. Nirvana-L= Nirvana Private Natural Reserve living collections, Valle del Cauca, Colombia, FairchildG = Fairchild Tropical Botanic Garden, FL, USA. Accessions to be vouchered when flowering are annotated with '*' and unvouchered with '**'. All the other herbarium acronyms are as found in the Index Herbariorum. When available, inaturalist observation number is provided in the locality field in squared brackets and the record can be accessed by replacing the number at the end of the following URL https://www.inaturalist.org/observations/ .

ID	Collector #	Species	Locality	Type of tissue	Collection
R3383	Skinner D. R3383	*Costus acreanus* (Loes.) Maas	Peru, Huanuco, Rio Derepente, Shinchi Roca [4618858]	F	BH
M10650	Maas P.J.M.10650	*Costus allenii* Maas	Colombia, Antioquia, road from Mutatá to Chigorodó, Finca Mejia.	F	MEDEL
R3077	Skinner D. R3077	*Costus amazonicus* – (Loes.) subsp. *amazonicus* J.F.Macbr	Ecuador, Zamora-Chinchipe, Guayzimi [4058104]	F	UC
M10732	Maas P.J.M. 10732	*Costus amazonicus* (Loes.) subsp. *amazonicus* J.F.Macbr	Colombia, Cauca, Mun. Santa Rosa, Vereda Santa Marta, Reserva Las Palmeras	F	CUVC
R3471	Skinner D. R3471	*Costus amazonicus* subsp. *krukovii* Maas	Brazil, Acre, Rio Azul [20860917]	F	inaturalist**

ID	Collector #	Species	Locality	Type of tissue	Collection
R3472	Skinner D. R3472	*Costus amazonicus* subsp. *krukovii* Maas	Brazil, Acre, Rio Azul [20861449]	F	inaturalist**
R3473	Skinner D. R3473	*Costus arabicus* X *scaber*	Brazil, Acre, Rio azul [20861871]	F	inaturalist**
M10691	Maas P.J.M. 10691	*Costus asplundii* (Maas) Maas	Colombia, Putumayo, Mocoa, old trail from Mocoa to San Francisco	F	COAH
R3338	Skinner D. R3338	*Costus asplundii* X *amazonicus* 'Pink Floyd'	Ecuador, Zamora-Chinchipe, Nangaritza Road [35227524]	F	FairchildG*
R3213	Skinner D. R3213	*Costus barbatus* Suess.	Costa Rica, San Jose, s of Rancho Redondo [3908511]	F	L, BH
R3368	Skinner D. R3368	*Costus barbatus* Suess.	Costa Rica, Cartago, Muñeco [3812159]	F	FairchildG*
R3361	Skinner D. R3361	*Costus bracteatus* Rowlee	Panama, San Blas, Nusagandi [35226734]	F	BH
R3315	Skinner D. R3315	*Costus comosus* (Jacq.) Roscoe	Mexico, Chiapas, Road to Morelos [3876879]	F	BH
R3307	Skinner D. R3307	*Costus erythrophyllus* Loes.	Colombia, Boyaca, Santa Maria [3883138]	F	L

ID	Collector #	Species	Locality	Type of tissue	Collection
M10673	Maas P.J.M. 10673	*Costus aff. erythrophyllus* Loes.	Colombia, Valle del Cauca, PNN Farallones de Cali	F	CUVC
R3469	Skinner D. R3469	*Costus erythrothyrsus* Loes.	Brazil, Acre, Rio Azul [20849621]	F	inaturalist**
R3470	Skinner D. R3470	*Costus aff. erythrothyrsus* Loes.	Brazil, Acre, Rio azul	F	-**
R3476	Skinner D. R3476	*Costus aff. erythrothyrsus* Loes.	Brazil, Acre, SESC [20865033]	F	inaturalist**
R3479	Skinner D. R3479	*Costus aff. erythrothyrsus* Loes.	Brazil, Acre, Rondon [20865033]	F	inaturalist**
R3176	Skinner D. R3176	*Costus fenestralis* Maas & H.Maas	Lyon Arb/West Africa--John Mood	F	L
M10646	Maas P.J.M. 10646	*Costus glaucus* Maas	Colombia, Antioquia, road from Mutatá to Chigorodó, Finca Mejia	F	MEDEL
R3489	Skinner D. R3489	*Costus guanaiensis* Rusby	Brazil, Acre, Rio Tarauaca [23607486]	F	FairchildG*
NIR1	s.n.	*Costus guanaiensis* var. *tarmicus* (Loes.) Maas	Colombia, Valle del Cauca, Reserva Natural Nirvana	F	Nirvana-L*

ID	Collector #	Species	Locality	Type of tissue	Collection
RA3353	Skinner D. RA3353	*Costus* hybrid unnamed	Panama, Colón, Santa Rita	F	BH
M10685	Maas P.J.M. 10685	*Costus laevis* Ruiz & Pav.	Colombia, Valle del Cauca, PNN Farallones de Cali, Colombia	F	CUVC
R3127	Skinner D. R3127	*Costus lasius* Loes.	Peru, Loreto, Urco Mirano [3995845]	F	L, BH
R3242	Skinner D. R3242	*Costus lasius* Loes.	Panama, Coclé, base of Cerro Gaital [3905513]	F	UC
M10654	Maas P.J.M. 10654	*Costus leucanthus* Maas	Colombia, Antioquia, along road from Mutatá to Dabeiba.	F	MEDEL
NIR2	s.n.	*Costus lima* K.Schum.	Colombia, Valle del Cauca, Reserva Natural Nirvana	F	Nirvana-L*
R3303	Skinner D. R3303	*Costus lima* K.Schum.	Colombia Choco, Bahia Solano [3882613]	F	FairchildG*
R3345	Skinner D. R3345	*Costus lima* K.Schum.	Ecuador, Bolivar, Caluma [3870799]	F	FairchildG*
R3362	Skinner D. R3362	*Costus lima* K.Schum.	Costa Rica, Cartago, CATIE [3813421]	F	FairchildG**

ID	Collector #	Species	Locality	Type of tissue	Collection
R3167	Skinner D. R3167	*Costus lima* X *comosus* 'Tico Tower'	Costa Rica, Puntarenas, Rio Rincon [4078153]	F	BH
R3298	Skinner D. R3298	*Costus lima* X *pulvurulentus* 'Reinaldo'	Costa Rica, Puntarenas, Rincon	F	BH
M10693	Maas P.J.M. 10693	*Costus longibracteolatus* Maas	Colombia, Putumayo, Mocoa, old trail from Mocoa to San Francisco	F	COAH
R1562	Skinner D. R1562	*Costus malortieanus* H.Wendl.	Cultivated plant of unknown origin	F	L
R3293	Skinner D. R3293	*Costus montanus* Maas	Costa Rica, UGA San Luis near Monteverde [3887623]	F	FairchildG*
R3371	Skinner D. R3371	*Costus montanus* Maas	Costa Rica, Acosta, Nacientes Palmichal [3811944]	F	FairchildG*
R3088	Skinner D. R3088	*Costus nitidus* Maas	Costa Rica, Limón, Reserva Hitoy-Cerere [4057806]	F	UC
R3295	Skinner D. R3295	*Costus pictus* D.Don 'Red Baron'	Costa Rica, Alajuela, Rio Penas Blancas [3887959]	F	BH
R3172	Skinner D. R3172	*Costus pictus* D.Don	Puerto Rico, Jayuja [38650949]	F	BH

ID	Collector #	Species	Locality	Type of tissue	Collection
R3314	Skinner D. R3314	*Costus pictus* D.Don	Mexico, Chiapas, Rio Cuilco, Cascada San Francisco [3877204]	F	BH
R3316	Skinner D. R3316	*Costus pictus* D.Don	Mexico, Chiapas, Manecal [3877097]	F	FairchildG*
R3317	Skinner D. R3317	*Costus pictus* X 'California Dreaming'	Mexico, Chiapas, Nueva California [35227725]	F	L, BH
R3211	Skinner D. R3211	*Costus plicatus* Maas	Costa Rica, Puntarenas, La Gamba	F	UC, L, BH
M10675	Maas P.J.M. 10675	*Costus plowmanii* Maas	Colombia, Valle del Cauca, PNN Farallones de Cali	F	CUVC
R3304	Skinner D. R3304	*Costus pulverulentus* C.Presl	Colombia, Choco, Bahia Solano [3882671]	F	FairchildG*
R3161	Skinner D. R3161	*Costus pulverulentus* X *villosissiumus* 'Rancho Sunrise'	Costa Rica, Palmichal, near P. N. La Cangreja [35228487]	F	BH
R3165	Skinner D. R3165	*Costus ricus* Maas & H.Maas	Costa Rica, Puntarenas, Cerro Nara [4108032]	F	UC
R3373	Skinner D. R3373	*Costus ricus* Maas & H.Maas	Costa Rica, Puntarenas, Oro Verde [4376418]	F	BH

ID	Collector #	Species	Locality	Type of tissue	Collection
M10638	Maas P.J.M. 10638	*Costus scaber* Ruiz & Pav.	Colombia, Antioquia, Mun. Carepa, Estación Biológica Tulenapa.	F	MEDEL
M10657	Maas P.J.M. 10657	*Costus scaber* Ruiz & Pav.	Colombia, Valle del Cauca, PNN Farallones de Cali	F	CUVC
M10694	Maas P.J.M. 10694	*Costus scaber* Ruiz & Pav.	Colombia, Putumayo, Mocoa, old trail from Mocoa to San Francisco	F	COAH
R3217	Skinner D. R3217	*Costus scaber* Ruiz & Pav.	Guyana, Prince Charles Trail	F	UC
R3384	Skinner D. R3384	*Costus scaber* Ruiz & Pav.	Peru, Huanuco, Rio Derepente [4623570]	F	BH
R3123	Skinner D. R3123	*Costus sinningiiflorus* Rusby	Peru, Loreto, Santa Cruz [3994151]	F	UC
M10670	Maas P.J.M. 10670	*Costus sp.*	Colombia, Valle del Cauca, PNN Farallones de Cali	F	CUVC
R3158	Skinner D.F_R3158	*Costus sp. alleni* X	Panama, Bocas del Toro, Rio Teribe [3982678]	F	UC
R3200	Skinner D. R3200	*Costus sp. nov.* Ecuador	Ecuador, Imbabura, Tulipe [3961679]	F	L
R3332	Skinner D. R3332	*Costus sp. nov.* Ecuador2	Ecuador, Zamora-Chinchipe, Podocarpus area [3869211]	F	FairchildG*

ID	Collector #	Species	Locality	Type of tissue	Collection
CUVCglab	s.n.	*Costus sp. nov.* Colombia	ex hort. Gardens of CUVC Herbarium, originally from Colombia, Valle del Cauca (glabrous form)	F	CUVC-LC*
CUVChirs	s.n.	*Costus sp. nov.* Colombia	ex hort. Gardens of CUVC Herbarium, originally from Colombia, Valle de Cauca, Buenaventura, El Oro (hirsute form)	F	CUVC-LC*
ME2018	s.n.	*Costus sp. nov.* Colombia	Colombia, Medellín, Gardens of MEDEL Herbarium	F	MEDEL-L
R3260	Skinner D. R3260	*Costus sp. nov.* Peru	Peru, Madre de Dios, Manu Learning Centre [3899521]	F	L
R3265	Skinner D. R3265	*Costus sp. nov.* Peru	ex hort. Wild collected in Peru, Madre de Dios, Pantiacolla (glabrous) [3899828]	F	UC
R3322	Skinner D. R3322	*Costus sp. nov.* Peru	Peru, Madre de Dios, Pantiacolla (pubescent) [3899828]	F	UC
M10733	Maas P.J.M. 10733	*Costus sp. nov.* Pitalito	Colombia, Putumayo, Between Mocoa and San Augustin	F	COAH
R3331	Skinner D. R3331	*Costus sp. nov.* Putumayo	Ecuador, Zamora-Chinchipe, Podocarpus area [3869140]	F	FairchildG*
R3218	Skinner D. R3218	*Costus spiralis* (Jacq.) Roscoe	Guyana, Iwokrama, Canopy Walk [3907004]	F	UC

ID	Collector #	Species	Locality	Type of tissue	Collection
R3252	Skinner D. R3252	*Costus spiralis* (Jacq.) Roscoe	Brazil, Mato Groso, Cristalino [3902217]	F	BH
R3264	Skinner D. R3264	*Costus vargasii* Maas & H.Maas	Peru, Madre de Dios, Pantiacolla [3899744]	F	BH
M10656	Maas P.J.M. 10656	*Costus villosissimus* Jacq.	Colombia, Valle de Cauca, Mun. Buenaventura, Alto Anchicayá	F	CUVC
20180711	s.n.	*Costus villosissimus* Jacq.	Colombia, Valle del Cauca, PNN Farallones de Cali	F	-**
R3074	Skinner D. R3074	*Costus villosissimus* Jacq.	Ecuador, Azuay, Andrate [4057858]	F	L
R3092	Skinner D. R3092	*Costus wilsonii* Maas	Costa Rica, Puntarenas, La Amistad [4003019]	F	BH, L
R3091	Skinner D. R3091	*Costus wilsonii* X *villosissimus* 'Mellow Yellow'	Costa Rica, Puntarenas, Reserva Durika [4079545]	F	BH
R3374	Skinner D. R3374	*Costus woodsonii* Maas	Costa Rica, Limón, Cahuita [4376423]	F	L
